# Versatile strategy using vaccinia virus-capping enzyme to synthesize functional 5′ cap-modified mRNAs

**DOI:** 10.1093/nar/gkad019

**Published:** 2023-02-03

**Authors:** Hirohisa Ohno, Sae Akamine, Megumi Mochizuki, Karin Hayashi, Shinichiro Akichika, Tsutomu Suzuki, Hirohide Saito

**Affiliations:** Center for iPS Cell Research and Application, Kyoto University, 53 Kawahara-cho, Shogoin, Sakyo-ku, Kyoto 606-8507, Japan; Center for iPS Cell Research and Application, Kyoto University, 53 Kawahara-cho, Shogoin, Sakyo-ku, Kyoto 606-8507, Japan; Graduate School of Medicine, Kyoto University, Yoshida-Konoe-cho, Sakyo-ku, Kyoto 606-8501, Japan; Center for iPS Cell Research and Application, Kyoto University, 53 Kawahara-cho, Shogoin, Sakyo-ku, Kyoto 606-8507, Japan; Center for iPS Cell Research and Application, Kyoto University, 53 Kawahara-cho, Shogoin, Sakyo-ku, Kyoto 606-8507, Japan; Department of Chemistry and Biotechnology, Graduate School of Engineering, The University of Tokyo, 7-3-1 Hongo, Bunkyo-ku, Tokyo 113-8656, Japan; Department of Chemistry and Biotechnology, Graduate School of Engineering, The University of Tokyo, 7-3-1 Hongo, Bunkyo-ku, Tokyo 113-8656, Japan; Center for iPS Cell Research and Application, Kyoto University, 53 Kawahara-cho, Shogoin, Sakyo-ku, Kyoto 606-8507, Japan

## Abstract

The potential of synthetic mRNA as a genetic carrier has increased its application in scientific fields. Because the 5′ cap regulates the stability and translational activity of mRNAs, there are concerted efforts to search for and synthesize chemically-modified 5′ caps that improve the functionality of mRNA. Here, we report an easy and efficient method to synthesize functional mRNAs by modifying multiple 5′ cap analogs using a vaccinia virus-capping enzyme. We show that this enzyme can introduce a variety of GTP analogs to the 5′ end of RNA to generate 5′ cap-modified mRNAs that exhibit different translation levels. Notably, some of these modified mRNAs improve translation efficiency and can be conjugated to chemical structures, further increasing their functionality. Our versatile method to generate 5′ cap-modified mRNAs will provide useful tools for RNA therapeutics and biological research.

## Graphical Abstract

**Graphical Abstract:**
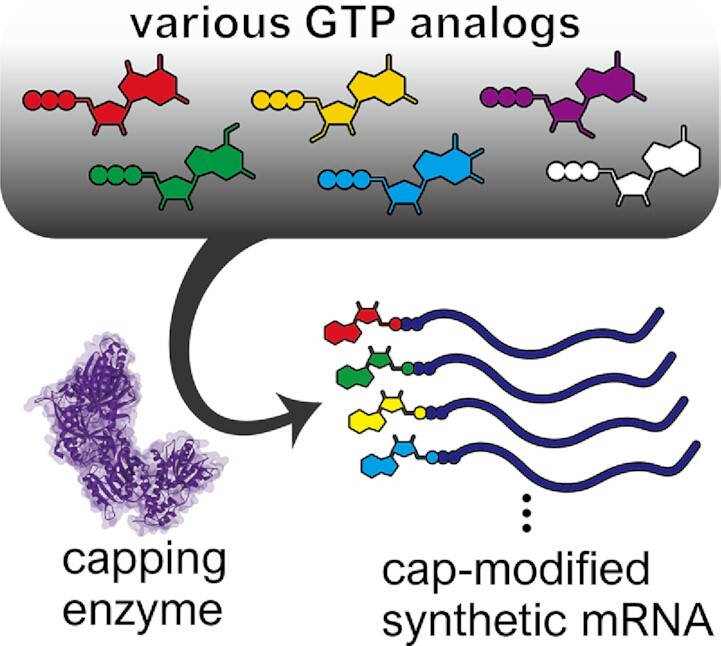
Vaccinia virus capping enzyme enables the synthesis of a wide variety of modified caps. Some of those caps can be expanded their chemical structure through bioconjugation technics.

## INTRODUCTION

Gene transfer using synthetic mRNA has steadily attracted attention for various biomedical applications (1–3). Unlike DNA or virus vectors, synthetic mRNA is less likely to be inserted into the genome, making it safer for gene and cell therapies. Additionally, synthetic mRNA can efficiently be transferred in the cytosol to produce proteins, because they do not need to enter the nucleus. Due to these characteristics, synthetic mRNAs are widely used for mRNA vaccines (4–7), genome editing (8,9), cell reprogramming (10,11) and cell isolation and separation (12,13) in cell therapies and regenerative medicine along with other applications.

However, RNA is chemically and biologically unstable, thus limiting its use for *in vivo* applications. mRNAs are degraded by various pathways in cells, but are also protected by their 5′ cap structure. In one degradation pathway, the cap is removed by a decapping enzyme, allowing the exonuclease to start degradation from the 5′-end (14). Chemical modification of the 5′ cap can block recognition by decapping enzymes to stabilize the mRNA. Additionally, by incorporating specific molecules at the 5′ cap, researchers can investigate the biological properties and expand the functionality of synthetic mRNA.

Currently, the most common method for preparing capped synthetic mRNA is *in vitro* transcription in the presence of a dinucleotide cap analog. In this strategy, a cap analog consisting of a dinucleotide connected by a 5′–5′ triphosphate bond is added to the transcription reaction. The analog competes with GTP for the first position of the transcript. Jemielity and colleagues have succeeded in producing mRNAs with improved translational activity and stability by synthesizing chemically-modified dinucleotides and using them as cap analogs (15–18). Consequently, the capped mRNAs can be produced in a one-step transcription reaction. However, the competition with GTP results in a fraction of synthetic mRNAs that are uncapped. Additionally, because the modified dinucleotides need to be synthesized chemically, many modified caps do not have widespread use.

**Figure 1.**
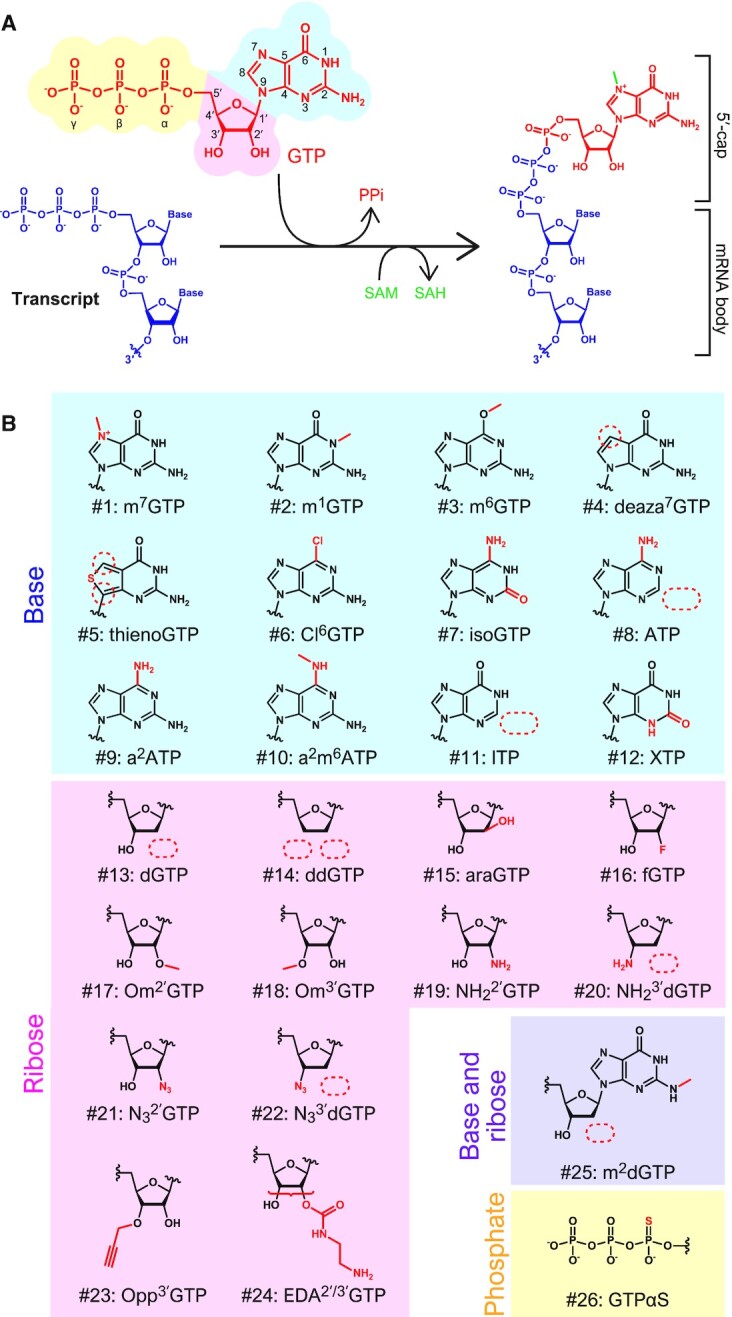
5′-cap modification by VCE. (**A**) The capping reaction catalyzed by VCE. VCE adds GTP (above, red) to the 5′ end of the transcript (left, blue) via triphosphate. Then, a methyl group is transferred from SAM (S-Adenosyl methionine, green) to the N7 of guanine. (**B**) Chemical structures of the GTP analogs used in this study. Modified moieties are indicated in red. See Table 1 for unabbreviated names.

An alternative method to synthesize 5′-capped mRNA is post-transcriptional enzymatic capping. After the synthesis of RNA that has a 5′ triphosphate through *in vitro* transcription, GTP is transferred to the 5′ end of the RNA by a capping enzyme (Figure 1A). We hypothesize that mRNAs with different chemically-modified 5′ caps could be efficiently synthesized by finding a capping enzyme that reacts with various GTP analogs instead of GTP. Studies report that a capping enzyme derived from Chlorella virus (PBCV-1) uses GTP analogs modified with bases or ribose for the 5′ end capping (19) (Table 1 and Supplementary Figure S1). However, the number of available GTP analogs is limited, and many of them have a lower capping efficiency compared to GTP (19).

**Table 1.** List of GTP analogs used for the enzymatic synthesis of modified 5′ cap structures

GTP analogs				VCE				PBCVCE
				GTase (GMP-incorporation)		MTase (N7-methylation)		GTase
Modified moiety	Abbreviation	Str. #	Name	Previous reports	This research	Previous reports	This research	[19]
Base	m^7^GTP	1	N7-Methylguanosine-5′-triphosphate	No [20]	Yes	-	-	No
	m^1^GTP	2	N1-Methylguanosine-5′-triphosphate	-	Yes	-	No	Yes
	m^6^GTP	3	O6-Methylguanosine-5′-triphosphate	-	Yes	-	No*	Yes
	s^6^GTP	27	6-Thioguanosine-5′-triphosphate	Yes [23]	-	Yes [23]	-	Yes
	ms^6^GTP	28	6-Methylthioguanosine-5′-triphosphate	-	-	-	-	Yes
	deaza^7^GTP	4	7-Deazaguanosine-5′-triphosphate	-	No	-	-	No
	thienoGTP	5	Thienoguanosine-5′-triphosphate	-	No	-	-	-
	oxo^8^GTP	29	8-Oxoguanosine-5′-triphosphate	-	-	-	-	No
	Br^8^GTP	30	8-Bromoguanosine-5′-triphosphate	-	-	-	-	Yes
	I^8^GTP	31	8-Iodoguanosine-5′-triphosphate	-	-	-	-	Yes
	Cl^6^GTP	6	2-Amino-6-chloropurineriboside-5′-triphosphate	-	Yes	-	No	Yes
	Cl^6^PRTP	32	6-Chloropurineriboside-5′-triphosphate	-	-	-	-	No
	isoGTP	7	Isoguanosine-5′-triphosphate	-	No	-	-	-
	ATP	8	Adenosine-5′-triphosphate	No [20]	No		-	No
	a^2^ATP	9	2-Aminoadenosine-5′-triphosphate	-	No	-	-	No
	a^2^m^6^ATP	10	N6-Methyl-2-aminoadenosine-5′-triphosphate	-	No	-	-	-
	ITP	11	Inosine-5′-triphosphate	-	Yes	-	Yes	Yes
	s^6^ITP	33	6-Thioinosine-5′-triphosphate	-	-	-	-	No
	ms^6^ITP	34	6-Methylthioinosine-5′-triphosphate	-	-	-	-	No
	XTP	12	Xanthosine-5′-triphosphate	-	No	-	-	No
	CTP	35	Cytidine-5′-triphosphate	No [20]	-	-	-	-
	UTP	36	Uridine-5′-triphosphate	No [20]	-	-	-	-
	RTP	37	Ribavirin-5′-triphosphate	Yes [22]		No [22]		
Ribose	dGTP	13	2′-Deoxyguanosine-5′-triphosphate	Yes [20]	Yes	Yes [20]	Yes	No
	d^3′^GTP	38	3′-Deoxyguanosine-5′-triphosphate	-	-	-	-	Yes
	ddGTP	14	2′,3′-Dideoxyguanosine-5′-triphosphate	-	No	-	-	No
	araGTP	15	Araguanosine-5′-triphosphate	-	Yes	-	Yes	-
	fGTP	16	2′-Fluoro-2′-deoxyguanosine-5′-triphosphate	-	Yes	-	Yes	Yes
	Om^2′^GTP	17	2′-O-Methylguanosine-5′-triphosphate	-	Yes	-	Yes	No
	Om^3′^GTP	18	3′-O-Methylguanosine-5′-triphosphate	-	Yes	-	Yes	Yes
	NH_2_^2′^GTP	19	2′-Amino-2′-deoxyguanosine-5′-triphosphate	-	Yes	-	Yes	-
	NH_2_^3′^dGTP	20	3′-Amino-2′,3′-dideoxyguanosine-5′-triphosphate	-	Yes	-	Yes	-
	N_3_^2′^GTP	21	2′-Azido-2′-deoxyguanosine-5′-triphosphate	-	Yes	-	Yes	-
	N_3_^3′^dGTP	22	3′-Azido-2′,3′-dideoxyguanosine-5′-triphosphate	-	Yes	-	Yes	-
	Opp^3′^GTP	23	3′-(O-Propargyl)-GTP	-	Yes	-	Yes	-
	Ant^3′(/2′)^GTP	39	3′(/2′)-O-Anthraniloyl-GTP	Yes [24]	-	Yes [24]	-	-
	Mant^3′(/2′)^GTP	40	3′(/2′)-O-(N-methylanthraniloyl)-GTP	No [24]	-	-	-	-
	DTB^2′^GTP	41	2′-Desthiobiotin-GTP	No [25]	-	-	-	-
	DTB^3′^GTP	42	3′-Desthiobiotin-GTP	Yes [25]	-	-	-	-
	EDA^2′/3′^GTP	24	2′/3′-O-(2-Aminoethyl-carbamoyl)-guanosine-5′-triphosphate	-	Yes	-	-	-
Base & ribose	m^2^dGTP	25	N2-Methyl-2′-deoxyguanosine-5′-triphosphate	-	No	-	-	-
	Ant^3′(/2′)^m^7^GTP	43	3′(or 2′)-(O-Anthraniloyl)-N7-methyl-GTP	Yes [24]	-	-	-	-
Phosphate	GTPαS	26	Guanosine-5′-O-(1-thiotriphosphate)	-	Yes	-	Yes	-

Results from this study and previous reports are summarized. The numbers in the column ‘Structure’ correspond to Figure 1b (#1-#26) and Supplementary Figure S1 (#27-#43). ‘Yes’, ‘No’, and ‘-’ mean ‘can be capped’, ‘cannot be capped’, and ‘not tested’, respectively. ‘3′(/2′)’ in #39, #40 and #43 indicates that molecules with a modification at 3′ (65%) or 2′ (35%) are included. See also ‘Discussion’ about MTase activity on #3 (m^6^GTP).

In order to discover capping enzymes with higher capping efficiency for various GTP analogs, a capping enzyme derived from the vaccinia virus capping enzyme (VCE) was investigated (20,21). VCE catalyzes three steps of the capping reaction including (1) hydrolysis of the 5′ triphosphate end of the RNA to diphosphate, (2) addition of a GMP molecule onto RNA diphosphate, which is transferred from GTP to form Gppp-RNA, and (3) SAM-dependent N7-methylation of the guanosine moiety to form m^7^Gppp-RNA (Figure 1A). VCE has been used to introduce GTP analogs to capture cap-binding proteins, such as fluorescent dyes or biotin (22–25) (Table 1). However, no comprehensive research has investigated the capping ability of VCE for the different GTP analogs, such as an azido-modified cap, for the purposes of engineering mRNAs.

In this study, we show that the VCE capping enzyme has a broad substrate selectivity that tolerates a greater variety of GTP analogs than others that have been previously tested (Table 1 and Figure 1B). We further confirm that several GTP analogs improve the translation efficiencies of the modified mRNAs in human cells. Additionally, we show that further modifications for bioconjugation can be easily added to the 5′ cap through utilizing azido-modified GTP analogs introduced by VCE.

## MATERIALS AND METHODS

### Materials

GTP analogs were purchased from TriLink BioTechnologies, except for m^7^GTP (Santa Cruz Biotechnology), dGTP (Toyobo), Opp^3′^GTP and EDA^2′/3′^GTP (Jena Bioscience). The ScriptCap m^7^G Capping System (CellScript) was used as a VCE.

### Validation of the capping efficiency by VCE

#### RNA preparation

To evaluate RNA capping efficiencies, a short RNA (5′-GGGCGAAUUAA-3′, 11 nt; Supplementary Table S1) with 5′ triphosphate was synthesized by *in vitro* transcription. For the template, two oligo DNAs (sense and anti-sense DNAs, see Supplementary Table S2) were annealed by heating and cooling in a hybridization buffer (10 mM Tris–Cl, 100 mM NaCl, pH 7.6). Using the template and MEGAshortscript (Thermo Fisher Scientific), a transcription reaction was performed overnight. To inhibit the production of undesired RNA due to run-off transcription, transcription was performed at 42°C. After treatment with TURBO DNase, the transcript was purified by denaturing PAGE (8.3 M urea, 0.5 × TBE, 20% polyacrylamide gel). Eluted RNA was extracted with phenol/chloroform and precipitated with ethanol. The concentration of recovered RNA was measured by using NanoDrop.

#### Capping reaction

For preparing short RNA, capping reactions were carried out using the ScriptCap m^7^G Capping System, which is a kit based on VCE, at a scale of 1/5 (20 μl), according to the manufacturer's protocol. For 200 pmol of RNA, 20 nmol of GTP analog and 8 U of ScriptCap Capping Enzyme (VCE) were incubated in ScriptCap Capping Buffer with 0.1 mM SAM and ScriptGuard RNase Inhibitor at 37°C. Half (10 μl) of the reaction solution was collected after 1 h, and the other half after 24 h. The reaction was stopped by the addition of Gel Loading Buffer II (Thermo Fisher Scientific) and was applied to denaturing PAGE (20% acrylamide, 8.3 M urea, 0.5 × TBE). After staining with SYBR Green II (Lonza), the gel was visualized using Typhoon FLA 7000 (GE Healthcare). The capping efficiency was calculated from the band intensity ratio of capped RNA and uncapped RNA using Image Quant (GE Healthcare). The experiment was repeated at least three times, and the mean values and standard deviations were calculated.

### Evaluation of N7-methylation efficiencies by mass spectrometry analysis

To evaluate the N7-methylation efficiencies, 11 nt short RNA was capped by VCE with each GTP analog. One nmol of short RNA was reacted with 100 nmol of GTP analog, 0.1 mM SAM, and 40 U of VCE at 100 μl scale. For the GTP analogs with lower capping efficiencies, the amounts of GTP analog were doubled (Om^2′^GTP [#17]) or the amounts of GTP analog and VCE were doubled (m^6^GTP [#3], ITP [#11]). The reaction was performed at 37°C for 1 h (GTP, m^1^GTP [#2], dGTP [#13], and Opp^3′^GTP [#23]) or 24 h (other analogs). To purify the capped RNA, denaturing PAGE was conducted. Eluted RNA was extracted with phenol/chloroform and precipitated with ethanol. The concentration of recovered RNA was measured by using NanoDrop.

For modified-capped short RNA analysis, the RNA samples (2 pmol) were mixed with 1/10 volume of 0.1 M triethylamine acetate (pH 7.0) and subjected to LC/nano ESI-MS on a Q Exactive Orbitrap mass spectrometer (Thermo Fisher Scientific) equipped with a splitless nanoflow high-performance LC (nano-HPLC) system (DiNa, KYA Technologies) using a nano-LC trap column (C18, 0.1  ×  0.5 mm, KYA Technologies) and a capillary column (HiQ Sil C18W-3, 0.1  ×  100 mm, KYA Technologies) as previously described (26,27). The RNA oligos were separated for 40 min at a flow rate of 300 nl/min by capillary LC using a linear gradient from 2 to 100% solvent B (v/v) in a solvent system consisting of 0.4 M 1,1,1,3,3,3-hexafluoro-2-propanol (HFIP) (pH 7.0) (solvent A) and 0.4 M HFIP (pH 7.0) in 50% methanol (solvent B). The eluent was ionized by an ESI source in a negative polarity and scanned over an *m/z* range of 600–2000. Xcalibur 2.0.7 (Thermo Fisher Scientific) was used for the system operation.

The LC/MS data were analyzed using the Xcalibur Qual browser (Thermo Fisher Scientific). In the negative mode of ESI, detection efficiencies of the RNA oligos having the same sequence but different modification statuses do not differ significantly because ESI ionization relies mainly on the numbers of phosphate groups, and not on the type of base modifications (28,29). Thus, the peak of each oligo was normalized by the sum of modified and unmodified oligos.

### *In vitro* decapping assay

Modified-capped short RNAs were prepared as described above. A-cap and G-cap short RNAs were co-transcriptionally synthesized using A-cap (G(5')ppp(5')A RNA Cap Structure Analog, New England Biolabs) or G-cap (GP3G (Unmethylated Cap Analog), JENA Bioscience) and purified by PAGE. For the DCP2 assay, 30 pmol of PAGE-purified RNAs were reacted with 50 U of mRNA Decapping Enzyme (New England Biolabs) in 1× MDE Buffer for 45 min at 37°C. For the DCPS assay, 30 pmol of PAGE-purified RNAs were reacted with 10 pmol of hDCPS (Recombinant human DCPS His Protein, Novusbio) in 10 mM Tris-OAc (pH 7.5), 100 mM KOAc, 2 mM Mg(OAc)_2_, and 2 mM DTT for 45 min at 37°C. Reactants were developed by denaturing PAGE. After staining with SYBR Green II (Lonza), the gel was visualized using Typhoon FLA 7000 (GE Healthcare). The decapping efficiency was calculated from the band intensity ratio of capped RNA and decapped RNA using Image Quant (GE Healthcare). Experiments were performed in triplicate, and the means and standard deviations were calculated. Statistical test was performed by Dunnett's test using KaleidaGraph (Synergy Software).

### Preparation of cap-modified mRNAs

The template for the *in vitro* transcription of humanized monomeric Azami-Green 1 (hmAG1) was made by PCR with KOD-Plus-Neo (TOYOBO) and a plasmid encoding the hmAG1. The PCR product was purified by a QIAquick PCR Purification Kit (Qiagen). Using the purified PCR product as a template, *in vitro* transcription reactions were performed using a MEGAscript T7 Transcription Kit (Thermo Fisher Scientific) at 37°C for 6 h. To suppress the immune response that occurs when RNA is introduced into cells, CTP and UTP were replaced by 5-methyl-CTP (TriLink) and pseudo-UTP (TriLink), respectively. The transcribed mRNA was treated with DNase and then purified using the RNeasy MinElute Cleanup Kit (Qiagen). mRNA capping with GTP analogs was performed using the ScriptCap m^7^G Capping System (CellScript) at a 1/5 reaction scale of that described in the manufacturer's protocol (20 μl). Fifty picomolar of mRNA, 20 nmol of GTP analog, 0.1 mM SAM, and 8 U of VCE were used. For the GTP analogs with lower capping efficiencies, the amounts of GTP analog were doubled (Om^2′^GTP [#17]) or the amounts of GTP analog and VCE were doubled (m^6^GTP [#3], ITP [#11]). The reaction was performed at 37°C for 1 h (dGTP [#13], m^1^GTP [#2] and Opp^3′^GTP [#23]) or 24 h (for other analogs). Because the capping efficiency varies depending on the GTP analog, uncapped mRNA was degraded and subsequently removed. The capping reactant was dephosphorylated by Antarctic Phosphatase (New England Biolabs) and phosphorylated by T4 polynucleotide kinase (Takara) to change the 5′ end of uncapped RNA to monophosphate from triphosphate. For 5000 ng of RNA, 10 U of Terminator 5′-Phosphate-Dependent Exonuclease (Epicentre), an enzyme that specifically degrades only 5′ monophosphorylated RNA, was added to degrade the uncapped mRNA. The reaction was conducted in Terminator Exonuclease Reaction Buffer A (Epicentre) for 1 h at 30°C. Then, column purification was performed to collect the capped mRNA.

The transfection control, iRFP670 mRNA, was transcribed by preparing a template for transcription in the same way as hmAG1 mRNA and adding a mixture of ARCA (Anti-reverse cap analog: TriLink) and GTP (4:1) instead of GTP. The transcript was treated with Antarctic Phosphatase and column purified.

### Validation of translational activity in cells

#### Cell culture

HeLa cells were maintained in DMEM High Glucose medium (Nacalai Tesque) supplemented with 10% FBS (Biosera), 1 × MEM Non-Essential Amino Acids Solution (Thermo Fisher Scientific) and 1 mM sodium pyruvate (Sigma). 293FT cells were maintained in DMEM High Glucose medium supplemented with 10% FBS, 1 × MEM Non-Essential Amino Acids Solution, 1 mM sodium pyruvate and 2 mM (1 ×) l-Glutamine (Thermo Fisher Scientific).

#### mRNA transfection

At 24 h before the transfection, HeLa cells were seeded onto 24-well plates at 0.5 × 10^5^ cells per well, and HEK293FT cells were seeded at 1 × 10^5^ cells per well. The prepared modified capped hmAG1 mRNA (200 ng) was transfected with iRFP670 mRNA (200 ng) using Lipofectamine MessengerMAX (Thermo Fisher Scientific). After 4 h, media was exchanged. The expression levels of fluorescent proteins were examined by a fluorescence microscope (IX81, Olympus) and flow cytometer (BD Accuri C6, BD Biosciences) 24 h after the transfection. The expression levels of hmAG1 were analyzed with flow cytometer. The flow cytometer results were analyzed using FlowJo (BD Biosciences). The expression level of hmAG1 was normalized by the expression level of co-transfected iRFP670. Experiments were performed in triplicate, and the means and standard deviations were calculated. Statistical test was performed by Dunnett's test using KaleidaGraph (Synergy Software).

### Time-course observation of protein expression level and mRNA level in cells

#### mRNA transfection and observation of protein expression

Modified-capped hmAG1 mRNAs were prepared as described above. At 24 h before transfection, HeLa cells were seeded onto 96-well plates at 1 × 10^4^ cells per well. The prepared modified-capped hmAG1 mRNA (50 ng) was transfected using Lipofectamine MessengerMAX (Thermo Fisher Scientific). After 4 h, media was exchanged. At 4, 24 and 48 h after mRNA transfection, the cells were observed by a fluorescence microscope (Cytell Cell Imaging System, GE Healthcare), and the expression levels of hmAG1 were analyzed by a flow cytometer (BD Accuri C6, BD Biosciences). The flow cytometer results were analyzed using FlowJo (BD Biosciences). Experiments were performed in triplicate, and the means and standard deviations were determined.

#### mRNA transfection, cDNA synthesis and real-time quantitative PCR (qPCR)

Modified-capped hmAG1 mRNAs were transfected to HeLa cells as described in the above ‘mRNA transfection and observation of protein expression’ section. Cells were harvested at 4, 24 and 48 h after mRNA transfection, and cDNA was synthesized using *SuperPrep* II Cell Lysis & RT Kit for qPCR (Toyobo) according to the manufacturer's protocol. In brief, after a rinse with PBS, cells in a 96-well plate were lysed by adding 60 μl of Lysis solution containing RNase Inhibitor and gDNA Remover and shaking for 10 min at room temperature. Eight microliters of the lysed solution was used for the 40 μl-scale reverse-transcription reaction (RT). RT was performed with 8 μl of 5× RT Master Mix by the following incubation program: 15 min at 37°C, 5 min at 50°C, then 5 min at 98°C. Two microliters of the RT reactant was used for 20 μl-scale real-time qPCR using THUNDERBIRD Next SYBR qPCR Mix (Toyobo) and QuantStudio3 (Thermo Fisher Scientific). For each RT sample, qPCR was performed in triplicate using primer sets for hmAG1 and GAPDH (Supplementary Table S3). The relative amount of hmAG1 mRNA was calculated by the Comparative Ct (ΔΔCT) method based on the averaged Ct values for 3 reactions of hmAG1 and GAPDH from the threshold automatically determined using QuantStudio Design & Analysis Software (Thermo Fisher Scientific). Amplification of a single product was confirmed by Melt Curve, and the size and purity of amplicons were also checked by capillary electrophoresis (Qsep100 DNA Fragment Analyzer, BIOptic). Experimental data were obtained from three biological replicates, and the means and standard deviations were determined.

### Bioconjugation

To confirm strain promoting azide-alkyne cycloaddition (SPAAC), 5′ UTR(1-11) RNA was prepared by capping with GTP, N_3_^2′^GTP (#21) or N_3_^3′^dGTP (#22) followed by PAGE purification. Two hundred picomoles of capped RNA (final concentration: 2.5 μM) was incubated with 100 nmol (1.25 mM) of DBCO-biotin (dibenzocyclooctyne-PEG4-biotin conjugate, Aldrich) or DBCO-AF647 (AF647 DBCO, Click Chemistry Tools) in 1x TBS (Tris-buffered saline: 50 mM Tris, 150 mM NaCl, pH 7.6) and 25% DMSO (dimethyl sulfoxide, Nacalai tesque) at 37°C for 24 h. The reactants were purified by ethanol precipitation and then applied to denaturing PAGE. The gel was stained with SYBR Green II and visualized using a Typhoon FLA 7000 (for AF647: 635 nm laser and R670 filter; for SYBR Green II: 473 nm laser and Y520 filter).

### mRNA visualization in HeLa cells

hmAG1 mRNA was capped with m^7^GTP (#1), N_3_^2′^GTP (#21) or N_3_^3′^dGTP (#22) using VCE and treated with Terminator exonuclease. Approximately 10 pmol of the mRNA was mixed with 10 000-fold DBCO-AF647 and reacted in 1× TBS, 25% DMSO for 1 h at 37°C. After the reaction, the unreacted dye was removed by purification with the Monarch RNA Cleanup Kit (New England Biolabs), and the concentration was measured using NanoDrop. mRNA labelling and the removal of unreacted dyes were confirmed by denaturing PAGE.

At 24 h before transfection, 0.5 × 10^5^ HeLa cells were seeded onto 24-well glass bottom plates (Greiner). Five hundred nanograms of fluorescently labelled or non-fluorescently labelled hmAG mRNA was transfected using Lipofectamine MessengerMAX (Thermo Fisher Scientific). After 4 h, cells were washed with PBS, stained with Hoechst 33342 (Thermo Fisher Scientific), and observed with a confocal quantitative image cytometer CellVoyager CQ1 (Yokogawa).

## RESULTS

### Enzymatic synthesis of cap-modified RNAs

**Figure 2.**
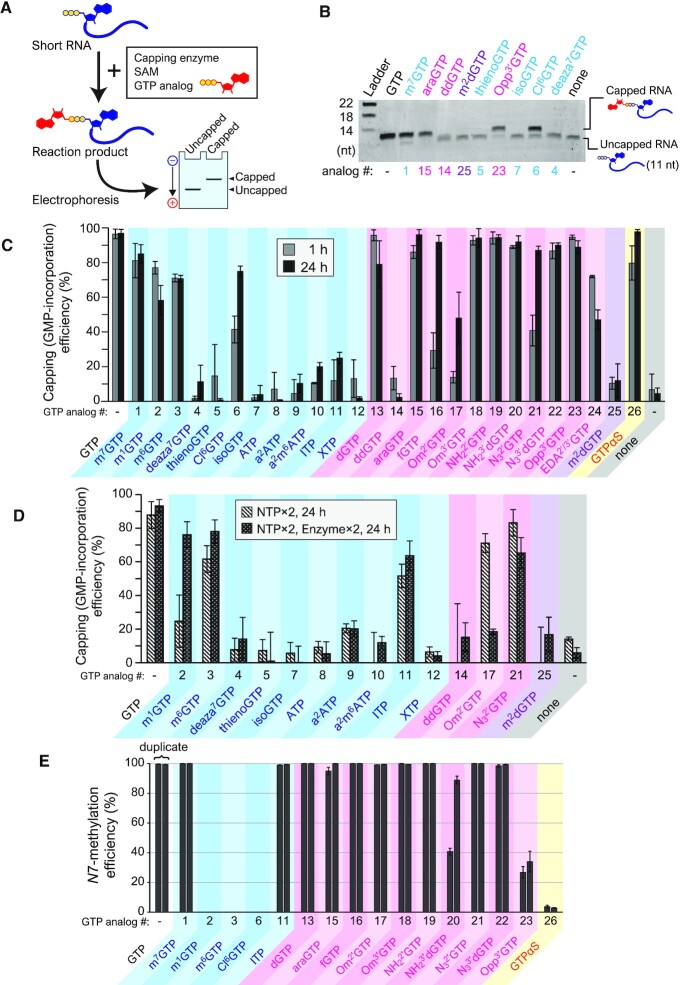
Capping with chemically-modified GTP analogs by VCE. (**A**) Outline of the experiment. Short RNA was reacted with VCE in the presence of a GTP analog and SAM. The reaction product was applied to electrophoresis to observe the capped and uncapped fractions. (**B**) A representative example of the results. Capped products were clearly distinguished from uncapped RNA. (**C**) Capping (GMP-incorporation) efficiencies of each GTP analog in standard conditions. Grey (left) and black (right) bars indicate the results for 1 h and 24 h reactions, respectively. (**D**) Capping efficiencies in modified conditions. (**E**) N7-methylation efficiencies analyzed with LC–MS.

First, we investigated whether various modified GTP analogs could be added to the 5′ end of RNA using VCE (Figure 1). Short RNAs that are 11 nucleotides long were transcribed *in vitro* and used to detect the addition of a GTP analog by gel mobility-shift assay (Figure 2A). After the capping reaction of short RNAs with VCE, SAM, and GTP analogs, the reactant was fractionated to capped or unreacted RNA by denaturing PAGE, and the GTP analog-incorporation efficiency was examined (Figure 2B shows an example).

The results of the reaction at 1 h are shown in Figure 2C (grey bars). Interestingly, many GTP analogs (e.g. dGTP [#13] and Om^3′^GTP [#18]) were efficiently conjugated with short RNA at a similar level to GTP (the original substrate). However, there were also some GTP analogs with low capping (GMP-incorporation) efficiency. After extending the reaction time to 24 h, the efficiency was improved for Cl^6^GTP (#6), fGTP (#16), Om^2′^GTP (#17), N_3_^2′^GTP (#21), and GTPαS (#26) (Figure 2C, black bars). Different conditions to further improve the capping efficiency were also tested. Doubling the GTP analog concentration enhanced the efficiency of Om^2′^GTP (#17) (Figure 2D). Moreover, by doubling both the VCE and GTP analog concentrations, the efficiencies of m^1^GTP (#2), m^6^GTP (#3), and ITP (#11) were also enhanced (Figure 2D), while other analogs, such as deaza^7^GTP (#4), ATP (#8) and XTP (#12), remained unchanged. Together, these observations confirmed that various kinds of GTP analogs can be added to the 5′ end of RNA using VCE.

VCE methylates N7 of GTP after forming Gppp-RNA in the presence of SAM. Thus, N7 methylation efficiencies of the incorporated GTP analog caps on the short RNA were analyzed with Liquid Chromatograph-Mass Spectrometry (LC-MS) (Figure 2E and Supplementary Figure S2). For base-modified analogs, m^1^GTP (#2), m^6^GTP (#3), and Cl^6^GTP (#6), the addition of methyl group was not detected at all. In contrast, ITP (#11) was efficiently methylated as with the natural substrate GTP. Most of the ribose-modified analogs were efficiently methylated. However, the methylation of NH_2_^3′^dGTP (#20) and Opp^3′^G (#23) was incomplete. GTPαS (#26) also showed a lower N7-methylation level. These results indicate that VCE also has broad substrate compatibility for N7-methylation, but the compatibility differs from that observed in capping activity (GMP-incorporation by guanylyltransferase (GTase) activity).

### Susceptibility of cap-modified RNAs to decapping enzymes

**Figure 3.**
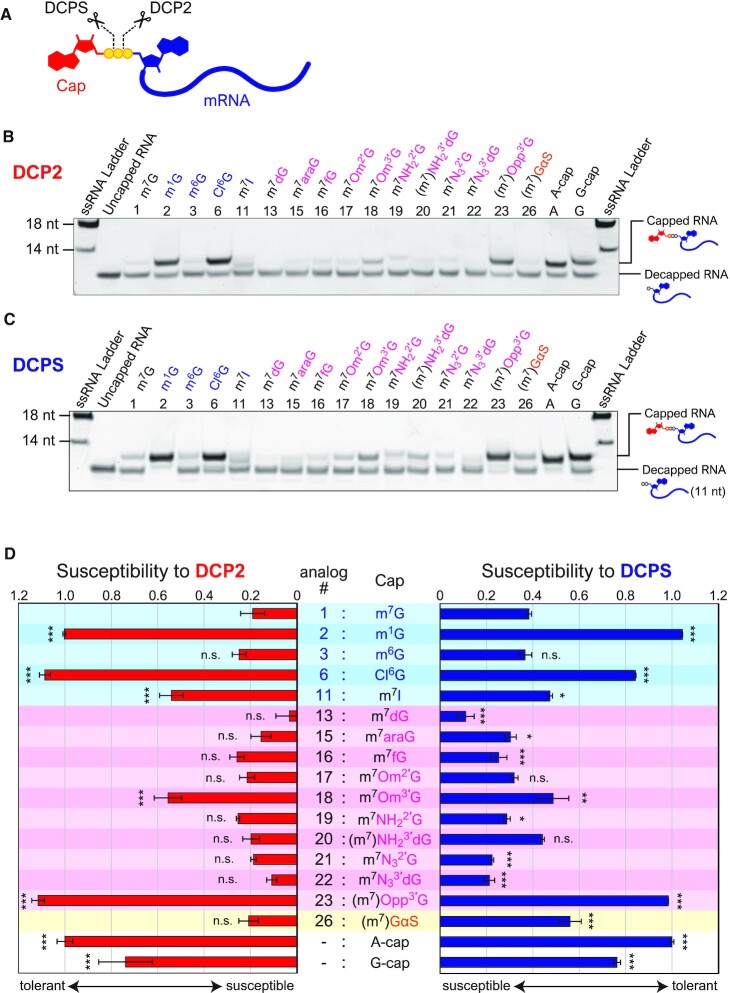
Susceptibility of cap-modified RNAs to decapping enzymes. (**A**) Decapping by Decapping enzymes. Cap structure is degraded by DCP2 and DCPS. Each decapping enzyme cleaves 5′-5′ triphosphate at a different position. (**B**) An example of decapping assay by DCP2. The lower and upper bands indicate the decapped RNA and remaining capped RNA, respectively. (**C**) An example of decapping assay by DCPS. (**D**) The sensitivities to decapping enzymes are shown based on the remaining amount of capped RNA. The values were normalized with the value of A-cap. Statistical significance to m^7^G-capped RNA (#1): n.s.: not significant, * *P* < 0.05, ** *P* < 0.01, *** *P* < 0.001 (Dunnett's test). m^7^; expected that the *N*7 is almost fully methylated; (m^7^): *N*7 is partialy methylated.

In eukaryote mRNA, the 5′ cap structure is detached by two types of decapping enzymes, DCP2 and DCPS. In the 5′→3′ decay pathway, DCP2 hydrolyses the 5′-5′ triphosphate bridging the cap and 5′-terminus of the mRNA and releases a m^7^G-diphosphate. The decapped mRNA with 5′-monophosphate is then degraded by the 5′→3′ exoribonuclease XRN1. DCPS hydrolyses the triphosphate bond between α and β phosphates in the remaining cap of the shortened RNA via 3′→5′ decay (Figure 3A) (14). Chemical modification of the 5′ cap structure may affect the interaction properties of the mRNA to these enzymes and its susceptibility to decapping reactions. Therefore, the susceptibility of the cap analog-modified RNAs against DCP2 (from yeast) and DCPS (from human) was examined (Figure 3B and C). The cap-modified RNAs were incubated with DCP2 or DCPS, and the remaining (capped) and decapped RNAs were analyzed with denaturing PAGE (Figure 3D). Although several cap-modified RNAs (#3, #13, #15–22) showed similar or reduced stability compared to natural m^7^G-capped RNA, it is noteworthy that the m^1^G (#2), Cl^6^G (#6) and (m^7^)Opp^3′^G (#23) modifications substantially increased RNA stability against both DCP2 and DCPS, which were comparable to A-cap and unmethylated G-cap (Figure 3D and Supplementary Figure S3). Additionally, m^7^Om^3′^G (#18) and (m^7^)GαS (#26) showed improved stability against DCP2 and DCPS, respectively, when compared with m^7^G-capped RNA.

### Translational activities of cap-modified mRNAs in cells

To investigate the translational activity of the cap-modified mRNAs for different GTP analogs in human cells, synthetic mRNAs were prepared. Because different capping efficiencies of each GTP analog potentially affect translational activity, capped RNA was purified by degrading uncapped RNA with a 5′ to 3′ exonuclease (see Methods and Supplementary Figure S4). Purified cap-modified mRNAs were transfected into HeLa or HEK293FT cells, and the expression levels of the green fluorescent reporter protein, humanized monomeric Azami-Green 1 (hmAG1), were analyzed using either a fluorescence microscope or flow cytometer. m^7^GTP (#1)-reacted RNA was used as the standard because its cap structure is the same as the natural 5′ cap, and m^7^G-capped mRNA was efficiently generated by VCE in the presence of m^7^GTP (Figure 2B, C). In addition, A-cap mRNA prepared by a dinucleotide cap analog was used as a negative control since it did not show cap-dependent translational activity.

**Figure 4.**
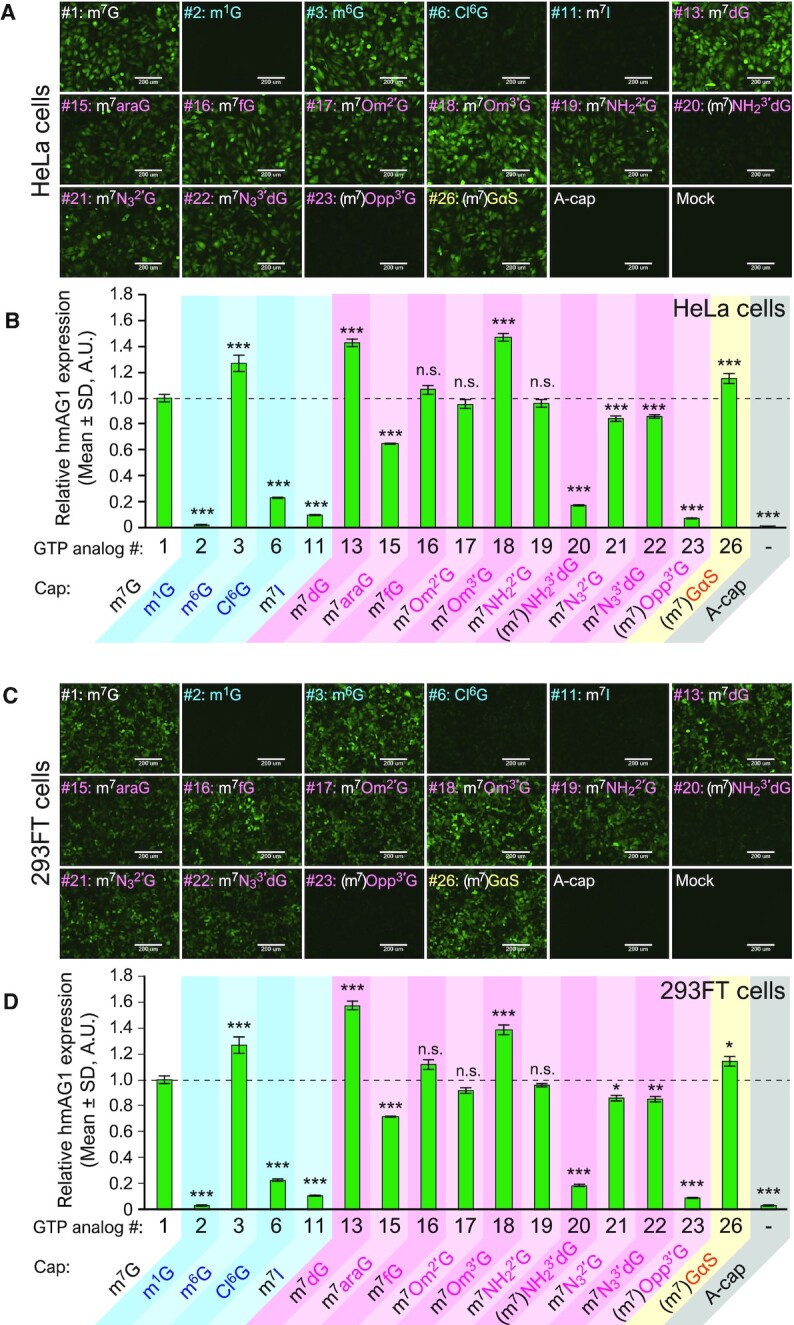
Translational activities of cap-modified mRNAs. (**A**) Fluorescence images of hmAG1 expressed from cap-modified mRNAs in HeLa cells. Scale bars, 200 μm. (**B**) The expression level of hmAG1 in HeLa cells as analyzed with flow cytometry. The values were normalized by the expression level of iRFP670 co-transfected as a transfection control. (**C**) Fluorescence images of hmAG1 expressed from cap-modified mRNAs in HEK293FT cells. Scale bars, 200 μm. (**D**) The expression level of hmAG1 in HEK293FT cells as analyzed with flow cytometry. The values were normalized by the expression level of iRFP670 co-transfected as a transfection control. Statistical significance to m^7^G-capped RNA (#1): n.s.: not significant, * *P* < 0.05, ** *P* < 0.01, *** *P* < 0.001 (Dunnett's test).

hmAG1 fluorescence was observed in most cap-modified mRNAs in HeLa cells (Figure 4A and Supplementary Figure S5). Figure 4b depicts the expression level of hmAG1 normalized by the co-transfected iRFP670 expression level (see also Supplementary Figure S6). The transfection efficiencies were similar across all tested cap-modified mRNAs (Supplementary Figure S6). A large decrease in the translation level was observed for mRNAs with m^1^G (#2), Cl^6^G (#6), m^7^I (#11), (m^7^)NH_2_^3′^dG (#20), or (m^7^)Opp^3′^G (#23) caps. m^7^fG (#16), m^7^Om^2′^G (#17) and m^7^NH_2_^2′^G (#19)-capped mRNAs showed comparable levels of translation as a natural 5′ cap. Interestingly, mRNAs with m^6^G (#3), m^7^dG (#13), m^7^Om^3′^G (#18) or (m^7^)GαS (#26) caps showed higher translation activity than those with a natural 5′ cap. Similar levels of translational efficiencies were observed in HEK293FT cells, indicating that the tendency is independent of the cell type (Figure 4C, D, Supplementary Figures S7 and S8). Thus, the translation efficiency can be tuned by synthesizing various cap analog-modified mRNAs with VCE.

**Figure 5.**
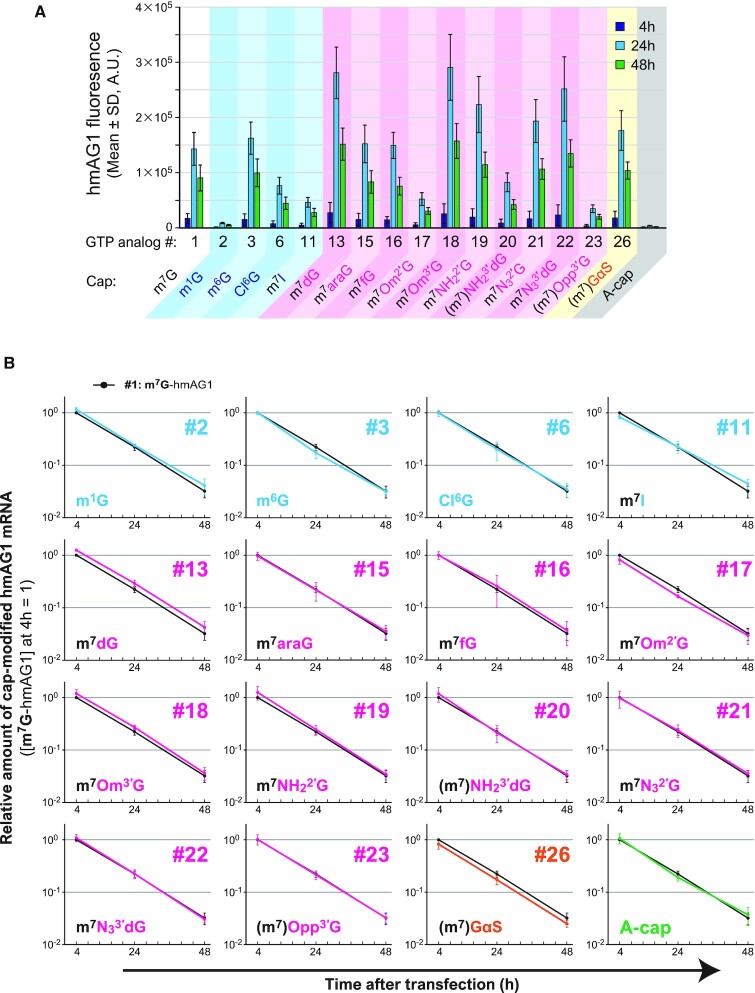
Time-course changes in expressed protein level and the amount of the residual mRNA in HeLa cells. (**A**) The expressed hmAG1 level was analyzed with flow cytometry. The mean of fluorescent intensity at each time point is shown. (**B**) The relative amount of residual mRNA after transfection was quantified by real-time qPCR. The black point indicates the mRNA level of natural m^7^G (#1)-capped mRNA. Each value was normalized to that of m^7^G (#1)-capped mRNA at 4 h.

We also investigated the time-course changes in protein expression and the amount of residual mRNA in HeLa cells (Figure 5 and Supplementary Figure S9). After 24 and 48 h of transfection, the cap-modified mRNAs with high translational activity consistently showed higher protein expression levels than the control m^7^G-capped mRNA. Among tested cap-modified mRNAs, there were no remarkable changes in either the rates of attenuation of protein expression levels or the decay rates of transfected mRNAs, as analyzed with either flow cytometry (Figure 5A) or real-time qPCR (Figure 5B), respectively.

### Bioconjugation

**Figure 6.**
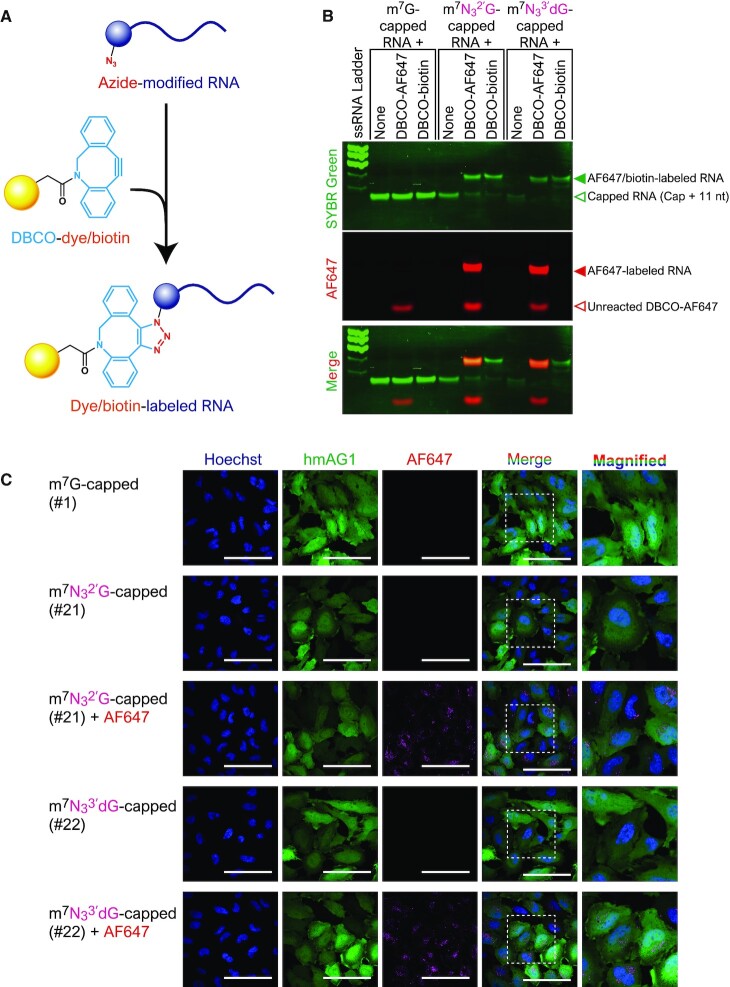
Bioconjugation using azide-containing modified caps. (**A**) The SPAAC reaction was used to introduce functional molecules to the azide group at the 5′ cap of RNA. (**B**) Fluorescent dye- or biotin-labelling of short RNA. Modified caps with the azide group were specifically labelled. (**C**) Confocal microscopic images of HeLa cells transfected with AF647-labelled hmAG1 mRNA. Scale bars, 100 μm. Images in the right columns are magnifications (100 μm × 100 μm) of the boxed regions in the adjacent columns.

Some GTP analogs introduced to the 5′ cap by VCE have useful functional groups such as a primary amino group, azide group or propargyl group (terminal alkyne) (#19–23). These functional groups are widely used for covalently linking functional molecules to biomolecules such as proteins and nucleic acids. Thus, we aimed to synthesize functionally-modified mRNAs by combining bioconjugation techniques with the presented VCE method. Biotin and a fluorescent dye were introduced into an azido-modified cap by a reaction between azido-alkyne, which is a typical type of click chemistry. The SPAAC reaction was used with dibenzocyclooctyne (DBCO) instead of terminal alkyne so that the reaction proceeded efficiently even in the absence of copper ion (30) (Figure 6A). After capping short RNA with GTP, N_3_^2′^GTP (#21), or N_3_^3′^dGTP (#22), the RNA reacted with DBCO conjugated with biotin or the fluorescent dye AF647 (Supplementary Figure S10a). As a result, it was confirmed that biotin or fluorescent dye can be added specifically to caps containing an azido group (Figure 6B).

Next, we investigated whether fluorescent AF647-cap-labelled mRNA synthesized by VCE and bioconjugation together could be used to visualize mRNA in living cells. Synthetic mRNA labeled with AF647 dye was prepared by the SPAAC reaction (Supplementary Figure S10b), and the labeled mRNA was transfected into HeLa cells. Confocal microscopy showed that the mRNA produced hmAG1 in the cells, and the red fluorescence of AF647 conjugated with the mRNA was seen in the cytoplasm in granular form, which is consistent with the pattern of transfected mRNAs (Figure 6C and Supplementary Figure S11). Thus, by combining the modified-cap method with bioconjugation, functional molecules could be introduced specifically at the cap site.

## DISCUSSION

### Enzymatic synthesis of cap-modified RNAs

In the present study, we demonstrate that VCE can attach many types of GTP analogs to the 5′-terminus of RNAs, making it possible to generate various functional mRNAs. In addition to the 26 varieties of analogs used here (Figure 1B), several studies have reported the possibility of capping by VCE with other GTP analogs (20,22–25) (Table 1). Based on the present and previous results shown in Table 1, modification of the base region seems to have a greater influence on VCE activity than changes to the ribose region. According to the crystal structure of the VCE and GTP complex, the guanine base of GTP is recognized by hydrogen bonds between O6 and Lys350, N7 and Lys350, and N2 and Asp390 and Thr239 (Supplementary Figure S12) (31). This explains why, in the case of modifications maintaining the hydrogen bonds (m^1^GTP [#2], m^6^GTP [#3] and Cl^6^GTP [#6]), the efficiency was lowered but capping (GMP-incorporation) was still possible (Figure 2C). Whereas, in the case of modifications in which the hydrogen bonds were disturbed, the capping reaction dropped greatly in efficiency (ITP [#11]) or was abolished completely (deaza^7^GTP [#4], thienoGTP [#5], isoGTP [#7], ATP [#8], a^2^ATP [9], a^2^m^6^ATP [10] and XTP [#12]) (Supplementary Figure S12).

Regarding the modification of ribose, the capping efficiency was decreased by 2′-hydroxyl group modifications such as fGTP (#16), Om^2′^GTP (#17), or N_3_^2′^GTP (#21). In contrast, GTP analogs with substituents at the 3′-OH positions, such as Om^3′^GTP (#18), NH_2_^3′^dGTP (#20), N_3_^3′^dGTP (#22), and Opp^3^′GTP (#23), showed similar capping efficiency as GTP. In the crystal structure of the VCE-GTP complex, the 2′-hydroxyl group of ribose is recognized by the hydrogen bond with Glu303, but the 3′-hydroxyl group is free (Supplementary Figure S12) (31). Considering this structure, in the case of a 2′ modification, the capping efficiency decreased most likely because of a disturbance in the hydrogen bond with Glu303 or steric hindrance. Whereas in the case of the 3′ modification, the capping efficiency was likely maintained due to the ribose binding with VCE without disturbing other bonds. Therefore, we anticipate that comparatively bulky substituents can be introduced at the 3′ position. Indeed, it was reported that a GTP analog modified at the 2′ position of ribose with desthiobiotin via a PEG linker could not be capped by VCE, but the same modification at the 3′ position did not affect the capping by VCE (25). ddGTP (#14) did not result in capped RNA, even under a modified condition. The 2′,3′-dideoxynucleotide is known to be prone to depurination (32). Therefore, it is possible that ddGTP-induced capping and N7-methylation may have in fact occurred, though with the m^7^G bases being subsequently removed (33), the reaction ultimately failed to produce any capped RNA.

Modification at the α-position phosphate group (thiophosphate) had little influence on capping by VCE. Therefore, other phosphate modifications, such as with boranophosphate, may also be allowed for capping. In this case, the oxygen atom at the α-position is substituted with BH_3_ and was previously reported to increase resistance to DCPS (34).

For N7-methylation (MTase) activity, ITP (#11) was completely methylated whereas m^1^GTP (#2) and Cl^6^GTP (#6) were not methylated at all. This suggests that N1 and O6 on the Watson-Crick edge may be important for base recognition (35). Furthermore, we also failed to observe methylation with m^6^GTP (#3). However, our LC-MS experiment could not distinguish between m^6^G and m^7^G due to their same mass. Therefore, there is a possibility that the m^6^G was converted to m^7^G by the N7-methylation followed by the removal of the methyl group at the O6 position. The cap structure created by VCE using m^6^GTP should be elucidated in future studies. As for ribose modification, high methylation efficiency was observed for most analogs, suggesting that the MTase activity is more tolerant to ribose modification than to base modification. Ribose modifications with lower methylation efficiency were observed for some 3′-modified nucleotides (NH_2_^3′^dGTP [#20] and Opp^3′^GTP [#23]), suggesting that, unlike capping (GTase) activity, the 2′-OH may be more permissive than the 3′-OH for MTase activity. Given that GTPαS (#26) was N7-methylated less efficiently, the phosphate group at the α-position (γ-position in capped mRNA) may also be important for recognition by the MTase domain of VCE. Although several crystal structures of VCE have been reported (31,36,37), there is no crystal structure of the MTase domain and 5′ cap complex. Determination of the structure may therefore uncover the detailed molecular mechanism for substrate selectivity of the MTase activity.

It has also been reported that the capping enzyme derived from Chlorella virus PBCV-1 can cap various GTP analogs, but not when dGTP (#13) or Om^2′^GTP (#17) were used as substrates (19) (Table 1). In contrast, it was shown here that VCE can cap with these two analogs. Additionally, the PBCV-1 capping enzyme has no MTase activity, but VCE can also methylate N7 using various GTP analogs.

### Translational activities of cap-modified mRNAs in human cells

In this study, it was revealed that some cap-modified mRNAs generated by VCE showed similar or better translational efficiencies compared with conventional mRNA capped with m^7^G (#1) (Figures 4 and 5A). For example, m^7^Om^3′^G (#18)-capped RNA exhibits higher translational activity (1.5- to 2-fold) compared with natural m^7^G (#1)-capped RNA in HeLa and HEK293FT cells. m^7^Om^3′^G (#18)-capped RNA has been reported to increase the translation level 1.5-fold in rabbit reticulocyte lysate or HEK293FT cells and improved stability of the RNA in the cells (15,19,38), which is consistent with our results. We first hypothesized that differences in mRNA cap stability might have contributed to the different translational levels observed because some cap analogs showed different susceptibilities against the decapping enzyme, DCP2 (Figure 3 and Supplementary Figure S3). Indeed, m^7^Om^3′^G (#18)-mRNA, which improved translation level, showed increased stability against DCP2 (Figure 3D). However, there was no clear correlation between the translation level of other analogs and susceptibility to the decapping enzymes. Moreover, observation of changes over time in protein translation and mRNA levels in HeLa cells showed no marked difference in the stability of mRNA with each cap analog (Figure 5). Under these experimental conditions, cap-independent mRNA degradation or cell division-mediated mRNA dilution may contribute more towards mRNA levels in a cell than the change in the stability against the decapping enzymes analyzed *in vitro*.

Interestingly, it was found that (m^7^)GαS (#26)-capped mRNAs showed high translational activity (Figure 4) although the N7 of guanine was only slightly methylated (Figure 2E). This unnatural cap-containing mRNA may be translated either with post-transfection methylation (N7-methylation may occur in cells after mRNA transfection), or without N7-methylation (eIF4E may recognize the analog without N7-methylation), or both. Phosphorothioate at the γ position, formed by capping with GTPαS (#26), has been reported to improve the affinity to eIF4E (39). It is likely that translation slightly improved due to this effect. However, the effect was moderate in this study, probably due to the low N7-methylation efficiency of GTPαS (#26) by VCE. To design mRNAs with higher translational activity, it may be useful to combine multiple modifications that have been demonstrated to induce translation. As the next challenge, it will be important to investigate whether GTP analogs with multiple modifications can be introduced into mRNA by VCE.

Notably, some analogs showed different results compared to previous studies. m^7^dG (#13) cap showed a translational level comparable with m^7^Om^3′^G (#18) cap in this study but was reported to have a lower translational efficiency than m^7^Om^3′^G-capped mRNA in rabbit reticulocyte lysate (15). Om^2′^-modification (#17) has also been reported to improve the translational activity in rabbit reticulocyte lysate and murine HC11 cells in previous studies (15,16), an effect that was not observed in the present study. We also observed a smaller or no enhancement in translational activity for m^7^fG (#16) cap than previously reported in HeLa cells (40). These different results may be caused by different types of evaluation systems (*in vitro* translation, different cell types and strains, etc.), reporter proteins, and 5′ UTR and 3′ UTR sequences. The lesser effect for (m^7^)Opp^3′^G (#23) as compared to another study (41) may also be caused by the low N7-methylation levels by VCE.

Several caps, such as m^1^G (#2), m^7^I (#11), and (m^7^)Opp^3′^G (#23) have little translational activity (Figure 4). However, RNAs capped with m^1^G or m^7^I have been reported to be more stable than uncapped RNA (19), which is consistent with our results against decapping enzymes (Figure 3d). Therefore, these caps could be used to protect the 5′ end of non-messenger RNA.

### Bioconjugation

This study showed that the 5′ cap with an azido group added by VCE can be easily labeled with fluorescent dye by the SPAAC reaction. In addition to fluorescent dyes and biotin, various alkyne-conjugated molecules are commercially available to introduce various molecules to the 5′ cap. SPAAC specifically reacts with a high functional group under a wide range of conditions. Additionally, it is possible to label synthetic RNA inside the cell (42).

Besides the VCE-based method reported here, an azido-modified dinucleotide cap analog and a guanine methylating enzyme have also been used to introduce an azido group to the 5′ cap region (42,43). In the former, the azido-modified cap analog should be incorporated at the 5′ end competitively with GTP during the transcription reaction so that RNA with the azido-modification at the 5′ cap can be prepared in a one-step reaction (42). However, since it is necessary to chemically-synthesize azido-modified dinucleotides, this method is not commonly used. In the other method, an analog in which the methyl group of SAM, which is a methyl group donor, is replaced with an azido group or a terminal alkyne and is synthesized and used as the substrate for trimethylguanosine synthase or methyltransferase, whereby the N7 or N2 position of guanine can be modified with the azido group or a terminal alkyne (43). However, more experimental steps are required as RNA with a 5′ cap needs to be synthesized and then treated with a methylating enzyme. It is also necessary to prepare guanine methylating enzymes and SAM analogs. Compared with those methods, the method presented here can utilize commercially available VCE and azido-containing GTP analogs to easily generate RNA possessing an azido group at the 5′ cap site with high efficiency by performing a one-step capping reaction.

In addition to the azido group, other functional groups available for bioconjugation can be introduced into the 5′ cap using VCE. The terminal alkyne (#23: Opp^3′^GTP) can be covalently bonded to a molecule having an azido group by the copper(I)-catalyzed azide-alkyne cycloaddition reaction (25). Primary amino groups possessed by NH_2_^2′^GTP (#19), NH_2_^3′^dGTP (#20) or EDA^2′/3′^GTP (#24) can also be used to covalently bond molecules conjugated with NHS-ester (44,45).

It has been reported that resistance to the decapping enzyme DCP2 is enhanced by adding a fluorescent dye to the 2′ or 3′ site of ribose or by introducing biotin at the 2′ site (46,47). Thus, further modification of the cap site by bioconjugation may be useful not only for imparting novel functions but also for improving RNA stability. There are various methods for modifying the RNA chain or 3′ end. Our method introduces the functional group orthogonal to those reactions at the 5′ site so that multiple site-selective RNA modifications may be possible. We believe that 5′ end modification by VCE will become a versatile and useful method for producing various types of functional RNAs in RNA engineering and nanotechnology fields (48,49).

In conclusion, we show that various cap-modified RNAs can be prepared easily and efficiently by using VCE and GTP analogs. Modified dinucleotide cap analogs are useful for synthesizing various modified-capped RNAs co-transcriptionally. However, it is necessary to chemically synthesize the dinucleotide cap analog to introduce rare modifications. In contrast, our VCE-based method can generate cap-modified mRNAs that exhibit various translation levels using commercially available materials without chemically-synthesized dinucleotides. Modified caps containing azide groups, amino groups, and alkynes can also be easily synthesized, making it possible to use this method as a 5′ end-specific modification method for RNA. This cap-modification method using the capping enzyme or the capping enzyme in combination with bioconjugation techniques will enable the generation of functional mRNAs with non-natural cap modifications and provide useful tools for the fields of RNA therapeutics and biological research.

## DATA AVAILABILITY

All data are available upon request.

## Supplementary Material

gkad019_Supplemental_File
