# Supplementary material for: Versatile strategy using vaccinia virus-capping enzyme to synthesize functional 5′ cap-modified mRNAs
Source: Nucleic Acids Res. 2023 Feb 3;51(6):e34. doi: 10.1093/nar/gkad019 (PMC10085709; doi:10.1093/nar/gkad019)
Supplement: gkad019_Supplemental_File [file gkad019_supplemental_file.pdf]

Supplementary Information

Versatile strategy using vaccinia virus-capping enzyme to synthesize functional 5′ cap-modified mRNAs

Hirohisa Ohno[1,†,*], Sae Akamine[1,2,†], Megumi Mochizuki[1], Karin Hayashi[1], Shinichiro Akichika[3], Tsutomu Suzuki[3] and Hirohide Saito[1*]

[1] Center for iPS Cell Research and Application, Kyoto University, 53 Kawahara-cho, Shogoin, Sakyo-ku, Kyoto, 606-8507, Japan

[2] Graduate School of Medicine, Kyoto University, Yoshida-Konoe-cho, Sakyo-ku, Kyoto, 606-8501, Japan

[3] Department of Chemistry and Biotechnology, Graduate School of Engineering, The University of Tokyo, 7-3-1 Hongo, Bunkyo-ku, Tokyo 113-8656, Japan

† Joint Authors

* To whom correspondence should be addressed. Tel: +81-75-366-7029; Fax:+81 75 366 7096; E-mail: saitou.hirohide.8a@kyoto-u.ac.jp

Correspondence may also be addressed to Hirohisa Ohno. E-mail: ohno.hirohisa.3u@kyoto-u.ac.jp

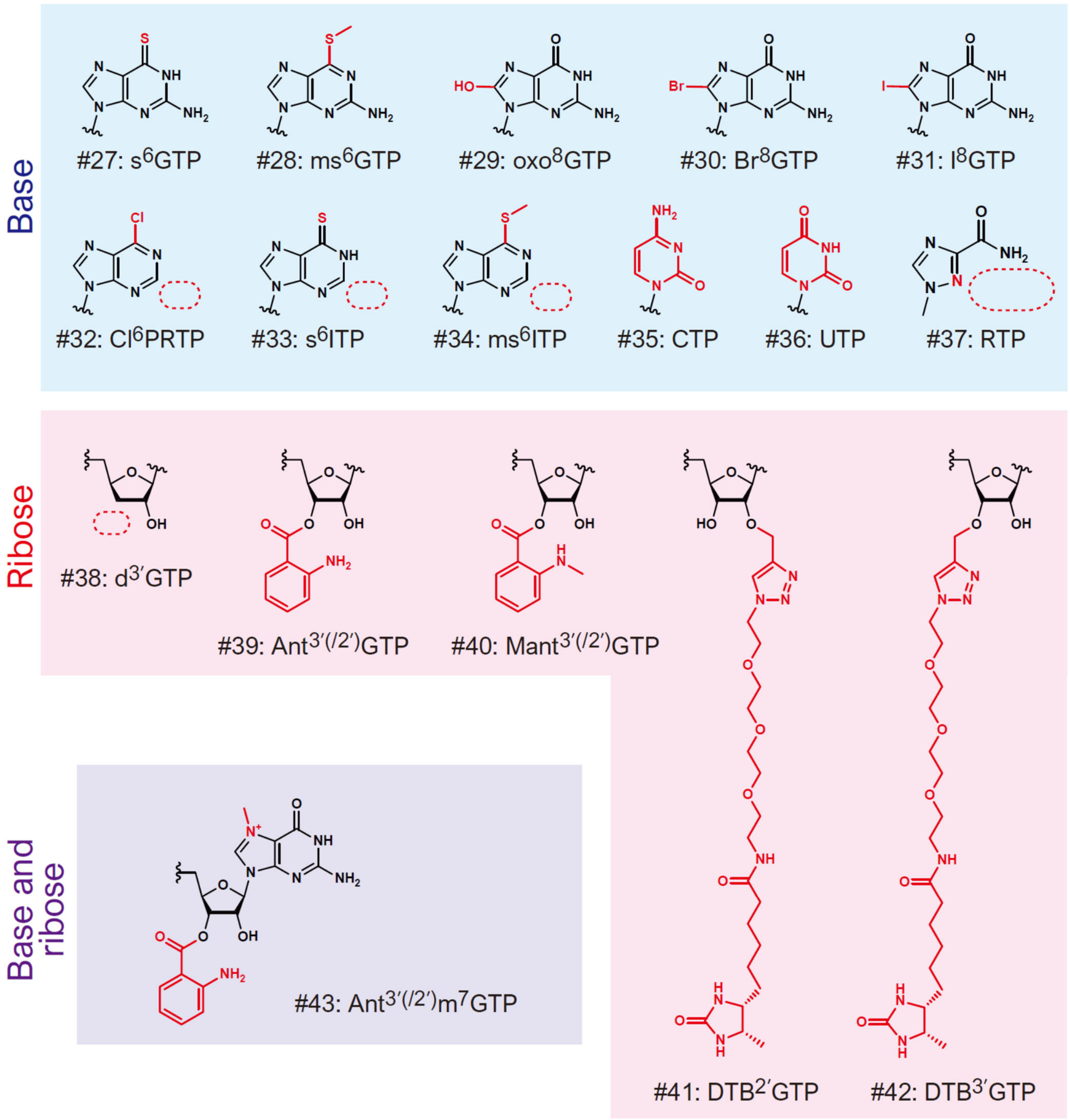

Supplementary Figure S1. Chemical structures of various GTP analogs used in previous reports. The numbers correspond to the numbers in Table 1. The positions of the struture different from GTP are shown in red.

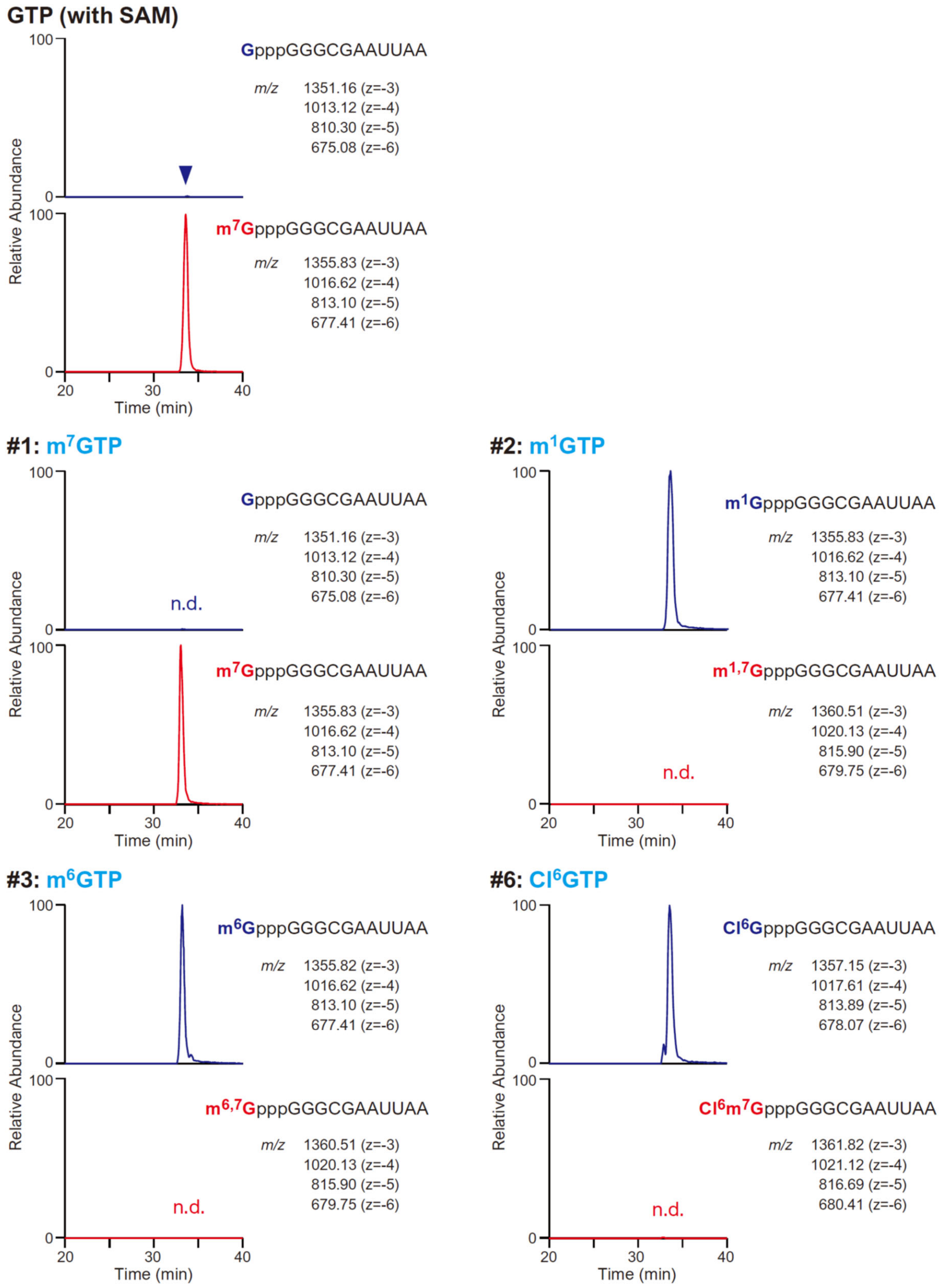

Supplementary Figure S2. Chromatograms of LC-MS analysis to evaluate N7-methylation efficiencies. Results from capping reaction using unmodified GTP and each modified GTP analogs are shown.

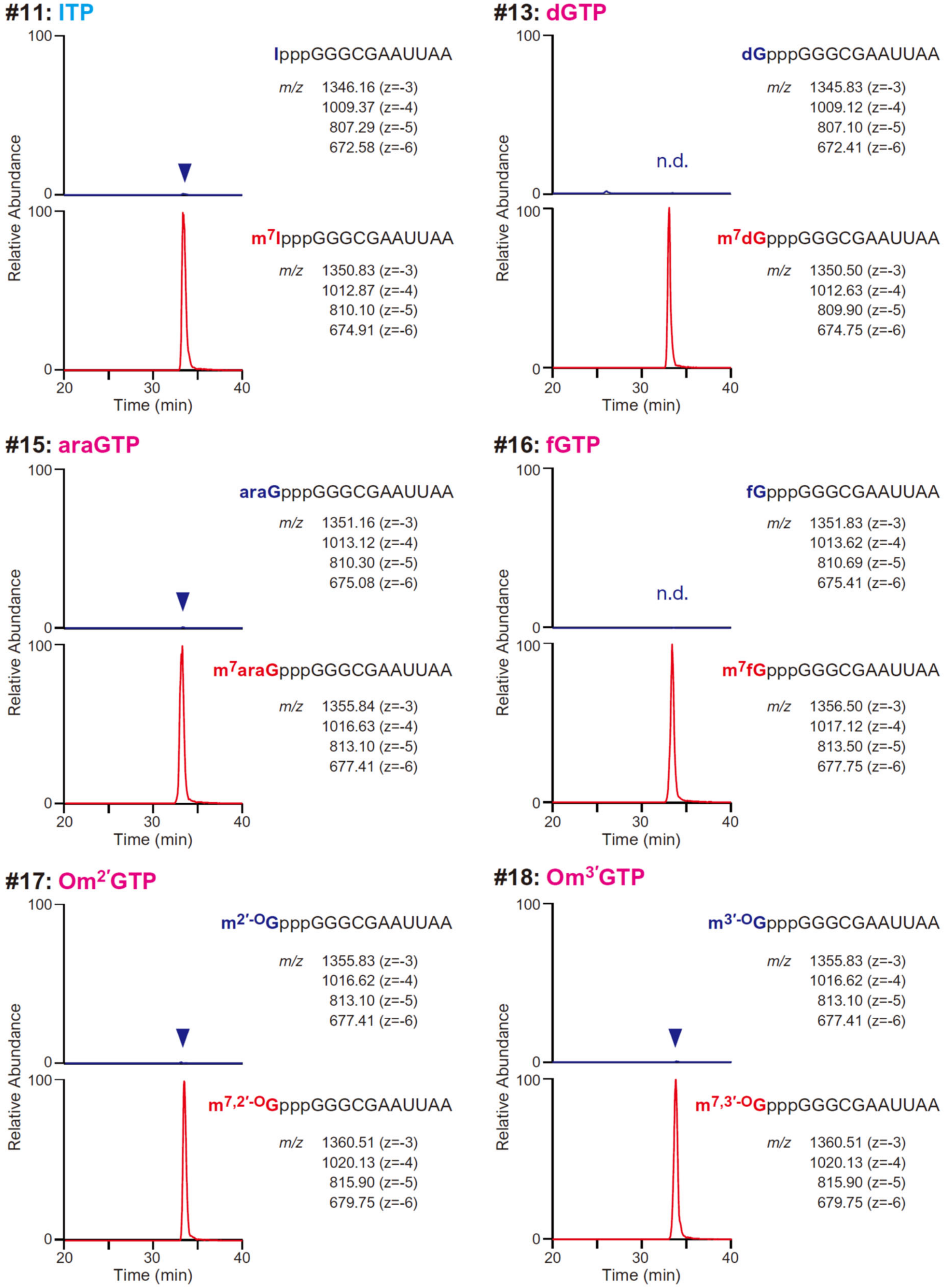

Supplementary Figure S2 (continued).

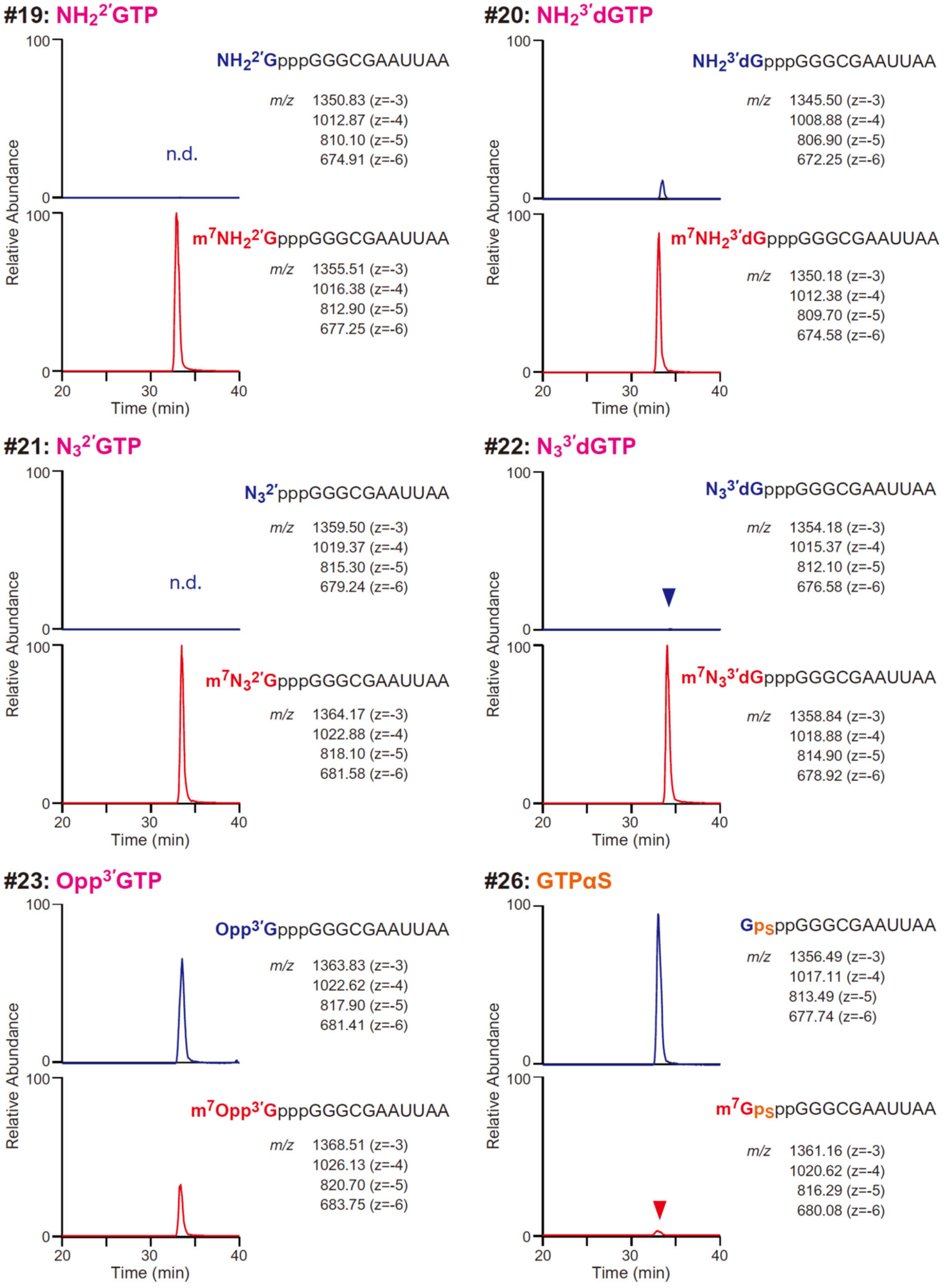

Supplementary Figure S2 (continued).

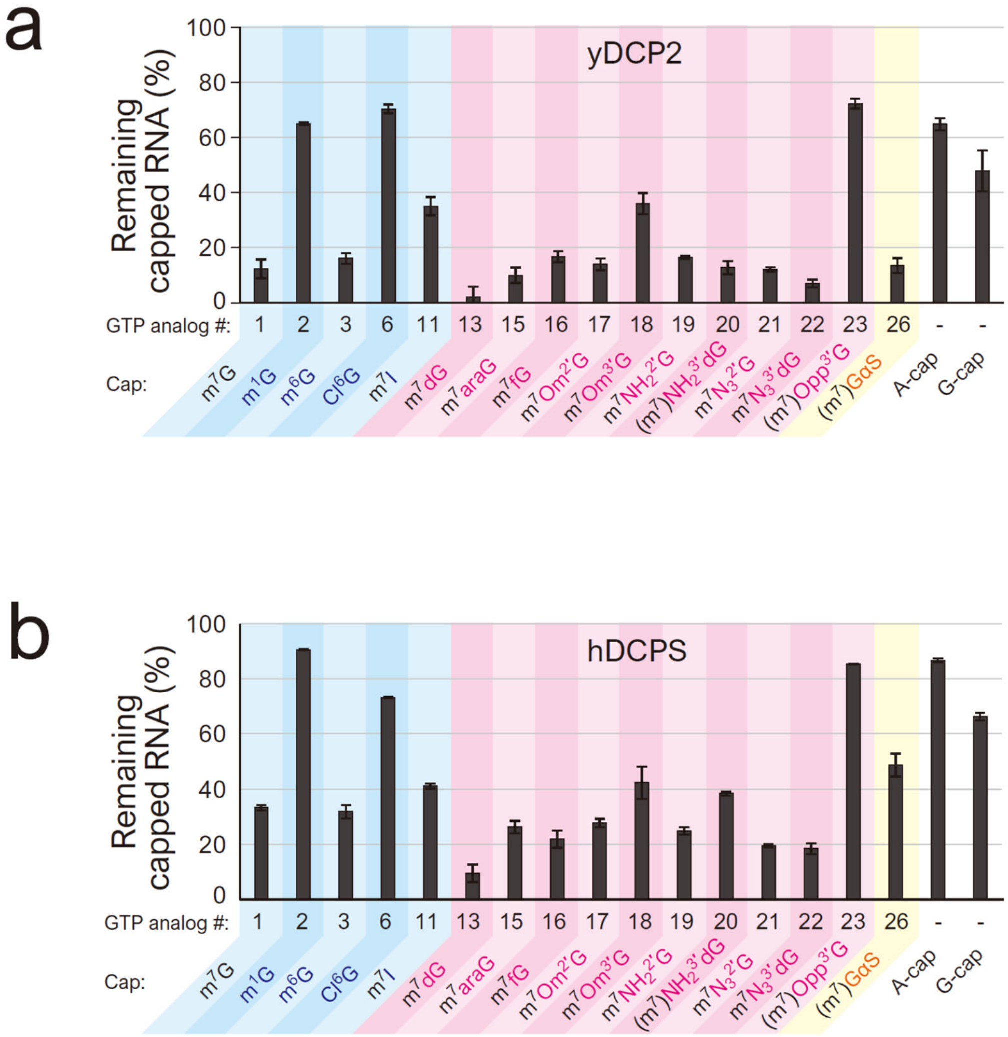

Supplementary Figure S3. *In vitro* decapping assay. After the decapping reaction, the amounts of decapped and capped RNA were quantified from each PAGE band intensity. (a) and (b) indicates the results of the assay using yeast DCP2 and human DCPS, respectively.

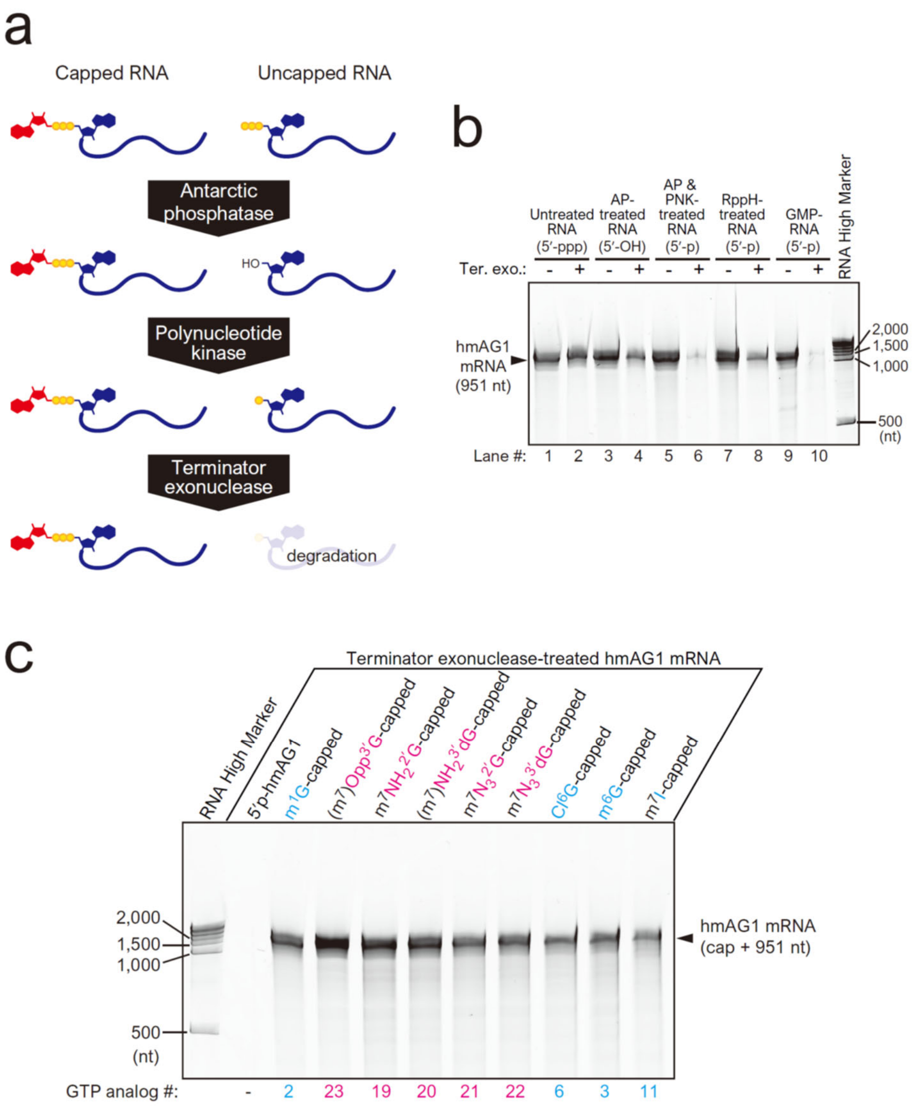

Supplementary Figure S4. Purification of capped mRNAs. (a) Scheme of the purification based on selective degradation of uncapped mRNA. Capping reactants including uncapped RNA were first treated with Antarctic phosphatase (AP) to dephosphorylate the 5′ terminal triphosphate of uncapped RNA. Next, polynucleotide kinase (PNK) phosphorylates the 5′ end of uncapped RNA. Then, uncapped RNA that has 5′ monophosphate was degraded by Terminator 5′-Phosphate-Dependent Exonuclease (Lucigen). **(b)** Terminator exonuclease treatment of uncapped mRNA. AP- and PNK-treated RNA that had 5′-monophosphate (5′-p) was almost completely degraded by the exonuclease. On the other hand, 5′-triphosphate (5′-ppp) RNA and 5′-hydroxyl (5′-OH) RNA were not degraded (Lanes 2 and 4). RppH (RNA 5′ Pyrophosphohydrolase), which removes pyrophosphate from the 5′-ppp, has been used to obtain 5′-p RNA. Therefore, we also tested RppH instead of AP and PNK (lanes 7 and 8). However, RppH-treated RNA showed less degradation than AP and PNK-treated RNA, implying the RppH activity was insufficient. GMP-hmAG1 (lanes 9 and 10) refers to 5′-p hmAG1 mRNA synthesized by *in vitro* transcription with GMP (guanosine-5′-p). It was also almost completely degraded by the exonuclease. (lane 10). **(c)** An example of Terminator purification of mRNAs capped with GTP analogs. The degradation efficiencies varied depending on the GTP analog used for capping and appeared to roughly correspond to the capping efficiency.

HeLa cells

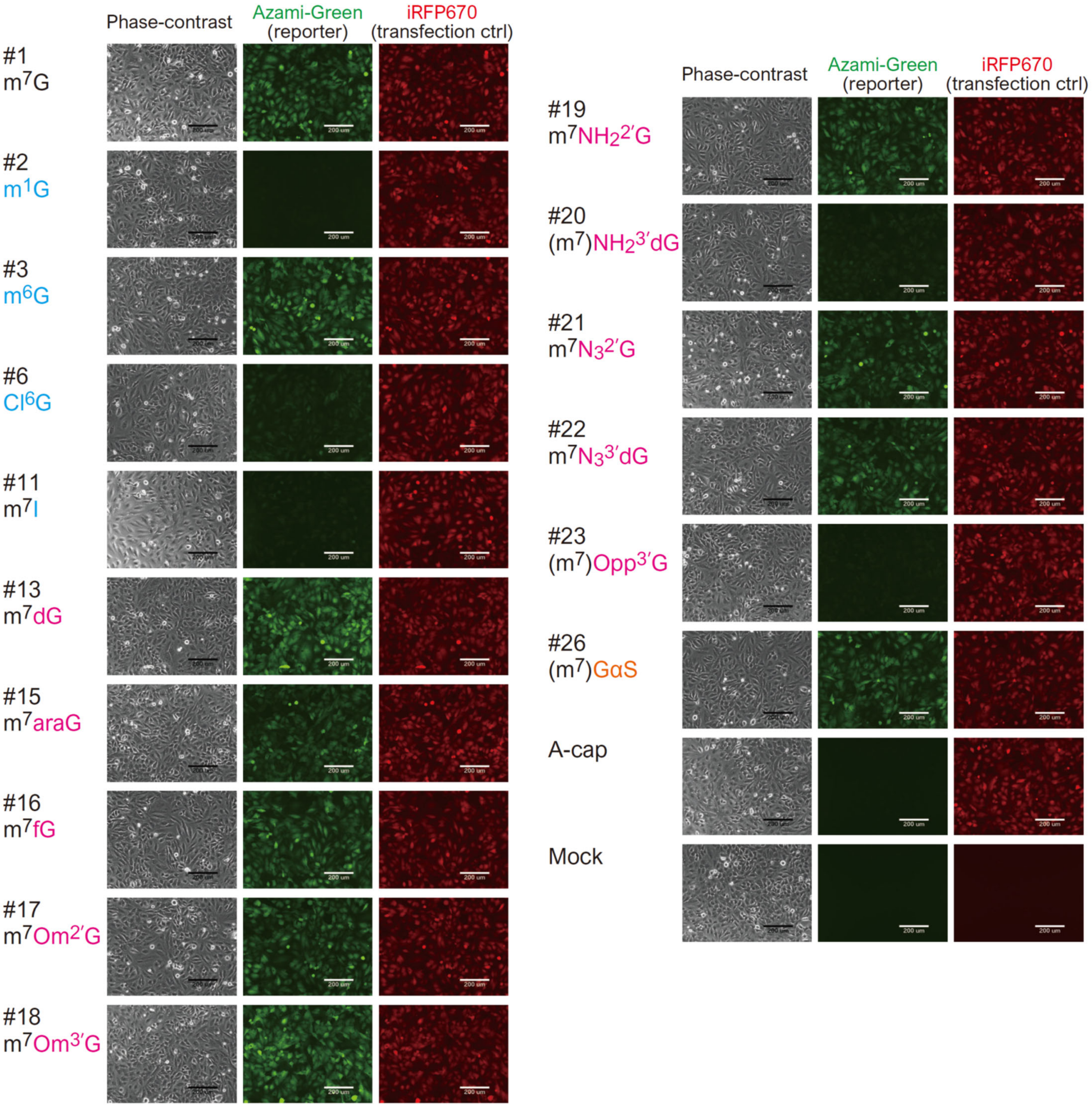

Supplementary Figure S5. Microscopic images of HeLa cells transfected with cap-modified mRNA. Green and red fluorescent images show the expression of Azami-Green (hmAG1) as a reporter and iRFP670 as a transfection control, respectively. "A-cap" refers to negative control hmAG1 mRNA with A instead of m^7G at the 5′ cap position. "Mock" indicates samples treated with transfection reagent alone and not treated, respectively. Scale bars, 200 μm.

HeLa cells

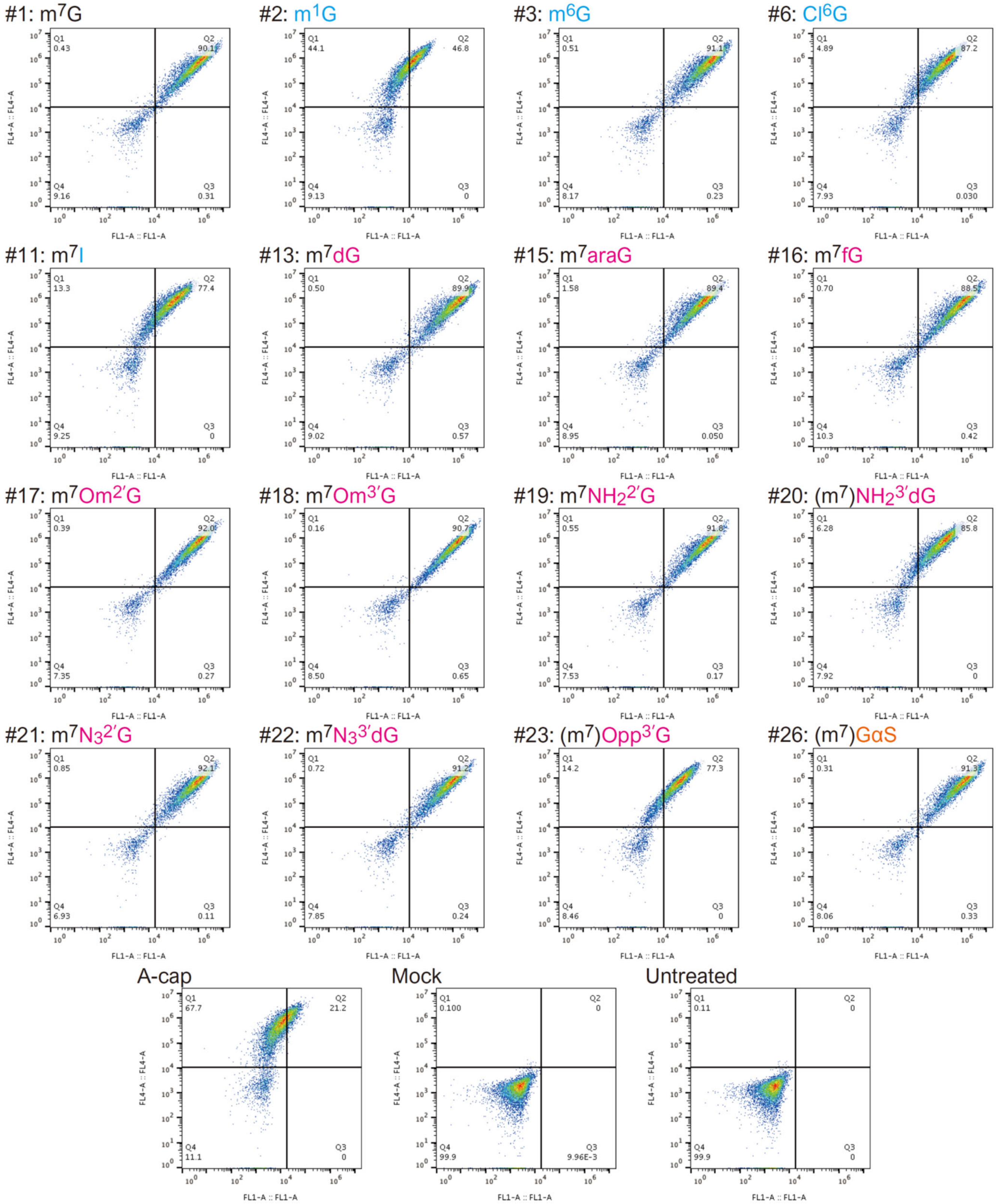

Supplementary Figure S6. Flow cytometric analysis of HeLa cells transfected with cap-modified mRNA. The vertical axis shows the fluorescence level of FL4, which corresponds to iRFP670 (reference), and the horizontal axis shows the fluorescence level of FL1, which corresponds to Azami-Green (hmAG1, reporter).

293FT cells

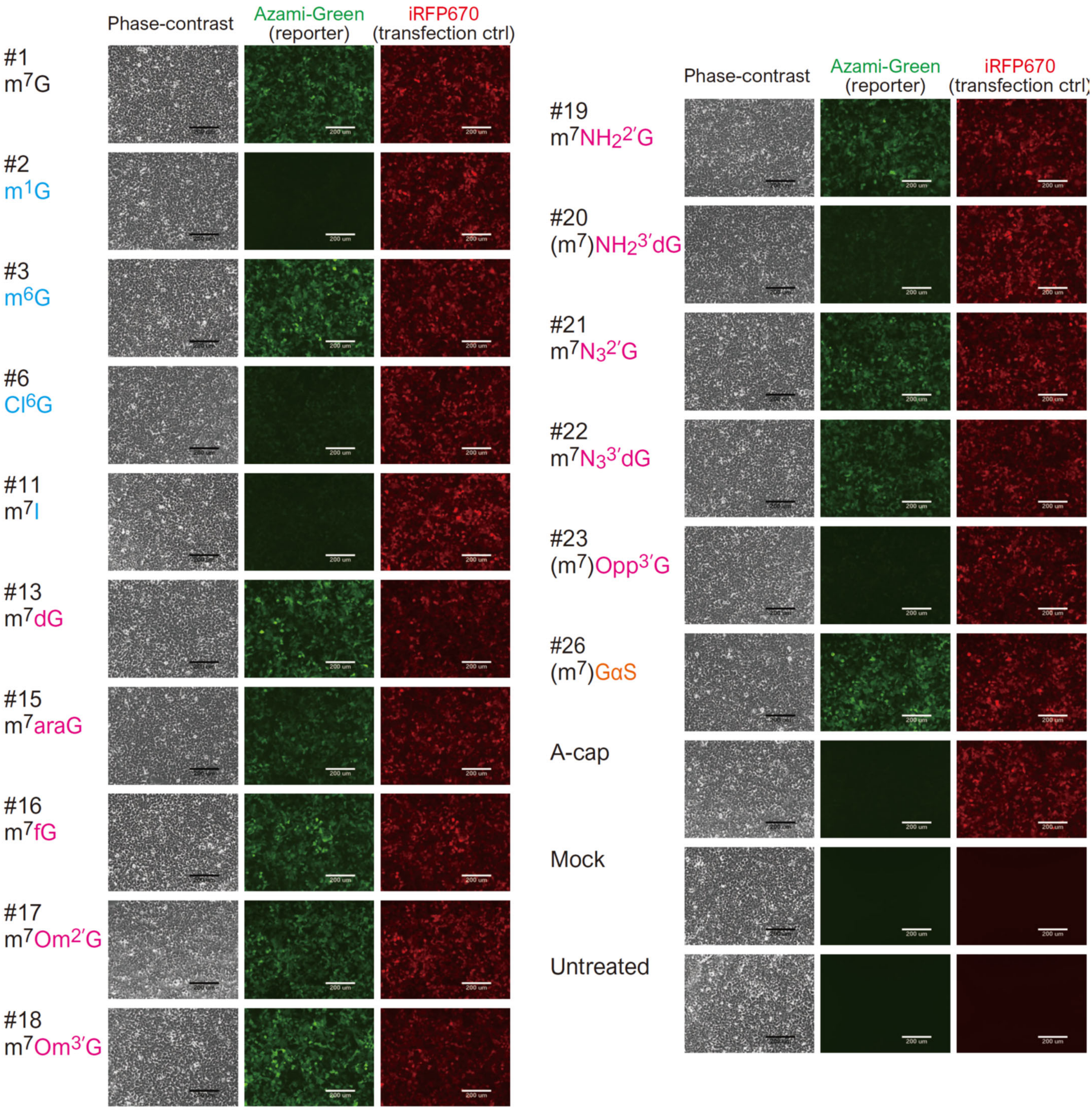

Supplementary Figure S7. Microscopic images of HEK293FT cells transfected with cap-modified mRNA. Green and red fluorescent images show the expression of Azami-Green (hmAG1) as a reporter and iRFP670 as a transfection control, respectively. “A-cap” means negative control hmAG1 mRNA with A instead of m^{7}G at the 5′ cap position. “Mock” indicates samples treated with transfection reagent alone and not treated, respectively. Scale bars, 200 μm.

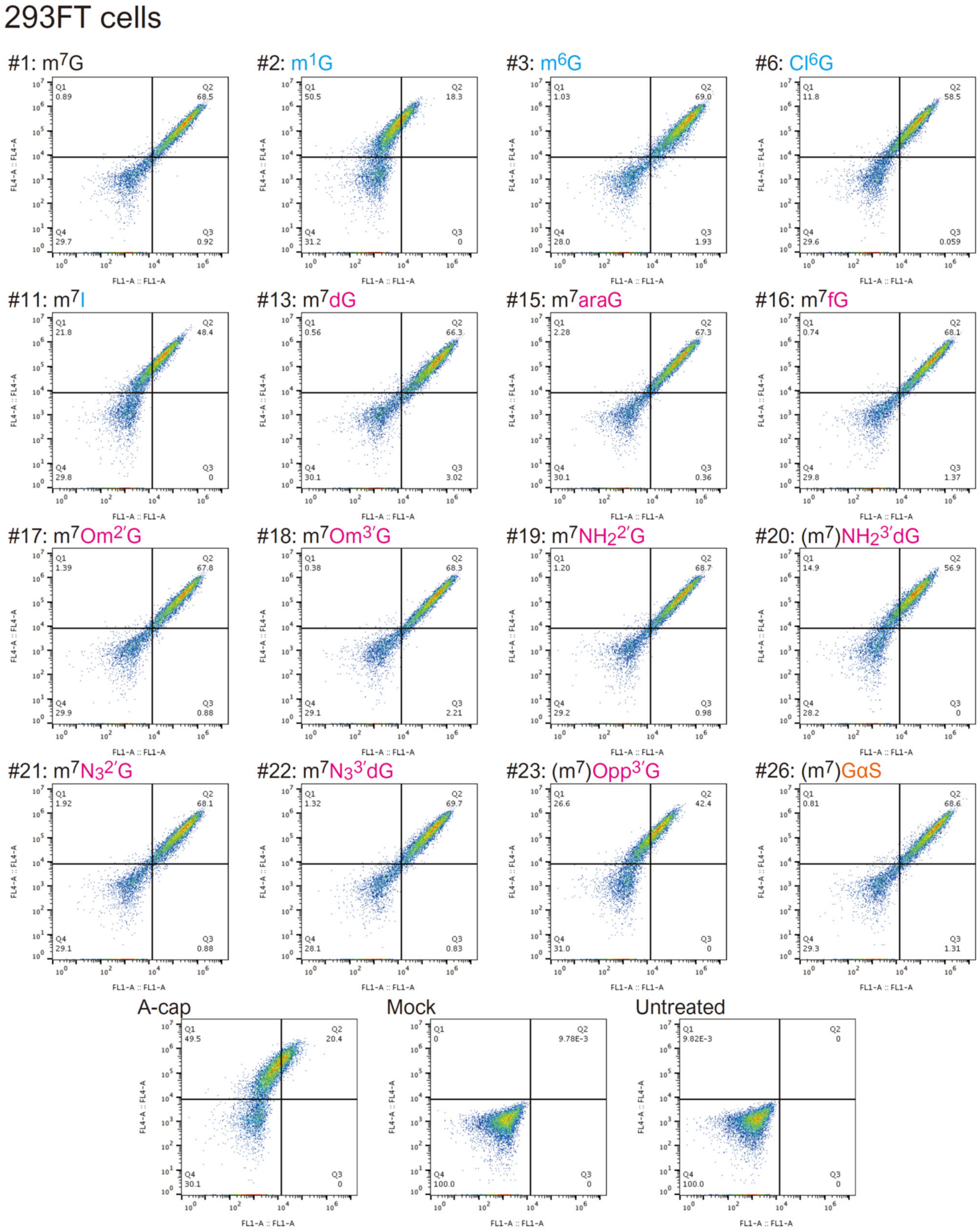

Supplementary Figure S8. Flow cytometric analysis of HEK293FT cells transfected with cap-modified mRNA. The vertical axis shows the fluorescence level of FL4, which corresponds to iRFP670 (reference), and the horizontal axis shows the fluorescence level of FL1, which corresponds to Azami-Green (hmAG1, reporter).

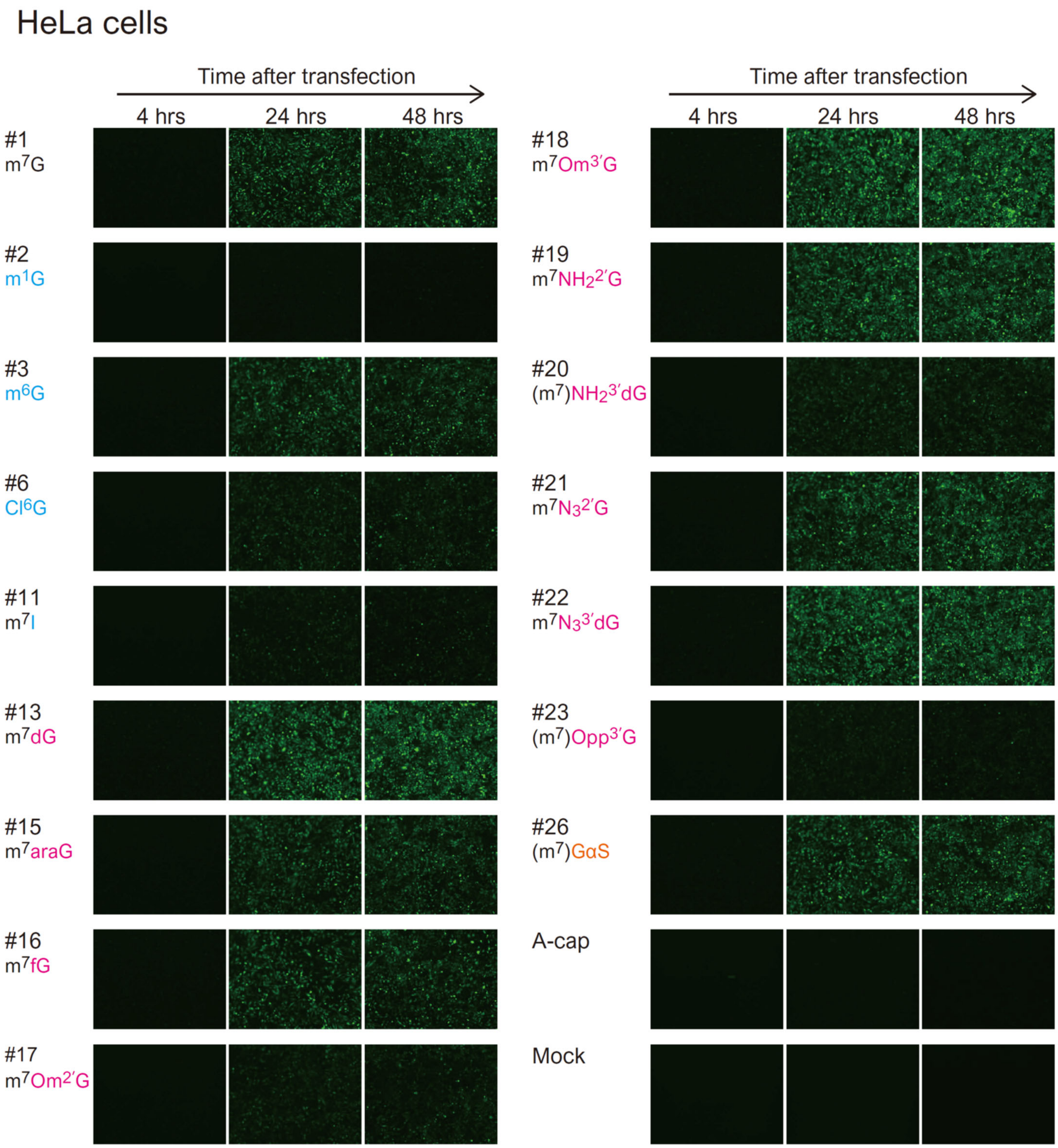

Supplementary Figure S9. Time-course observation of protein expression in HeLa cells. The fluorescence of hmAG1 expressed from cap-modified mRNA was observed at each time point after transfection.

a

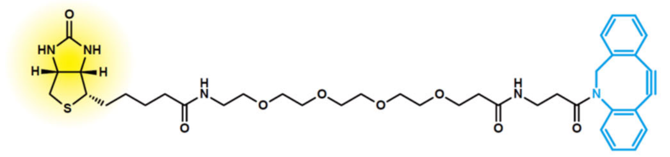

DBCO-biotin (Dibenzocyclooctyne-PEG4-biotin conjugate)

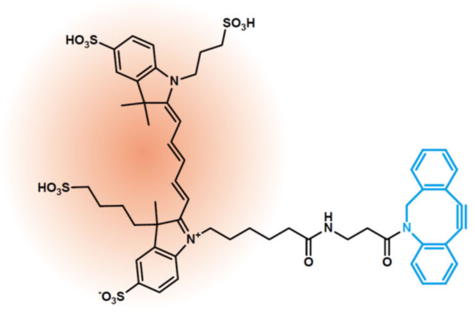

DBCO-AF647 (AF 647 DBCO)

b

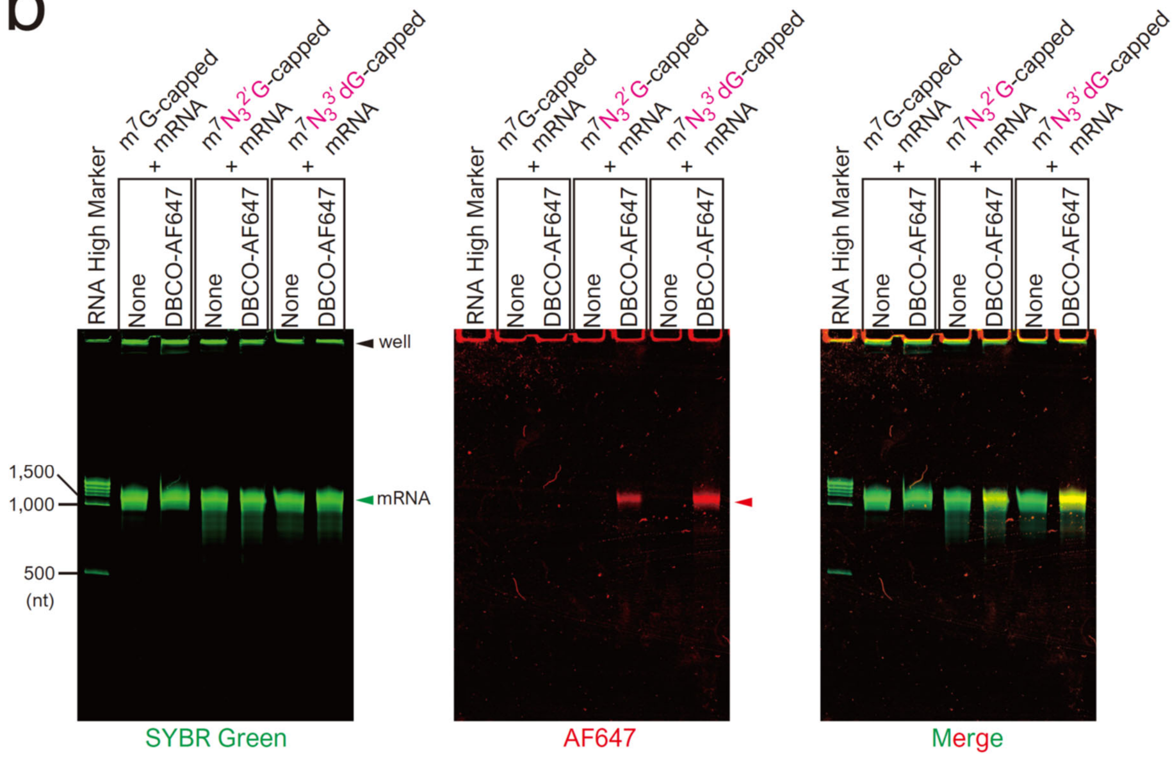

Supplementary Figure S10. Cap-specific labelling using a click chemistry reaction between an azide-containing cap and DBCO (dibenzocyclooctyne) derivatives. (a) Chemical structures of DBCO-biotin and DBCO-AF647. **(b)** Fluorescent labelling of the 5′ cap of mRNA. hmAG1 mRNAs capped with GTP, $N_3^{2'}$GTP (#21) or $N_3^{3'}$dGTP (#22) and purified using Terminator exonuclease were incubated with or without DBCO-AF647. Azide group-specific labelling was observed.

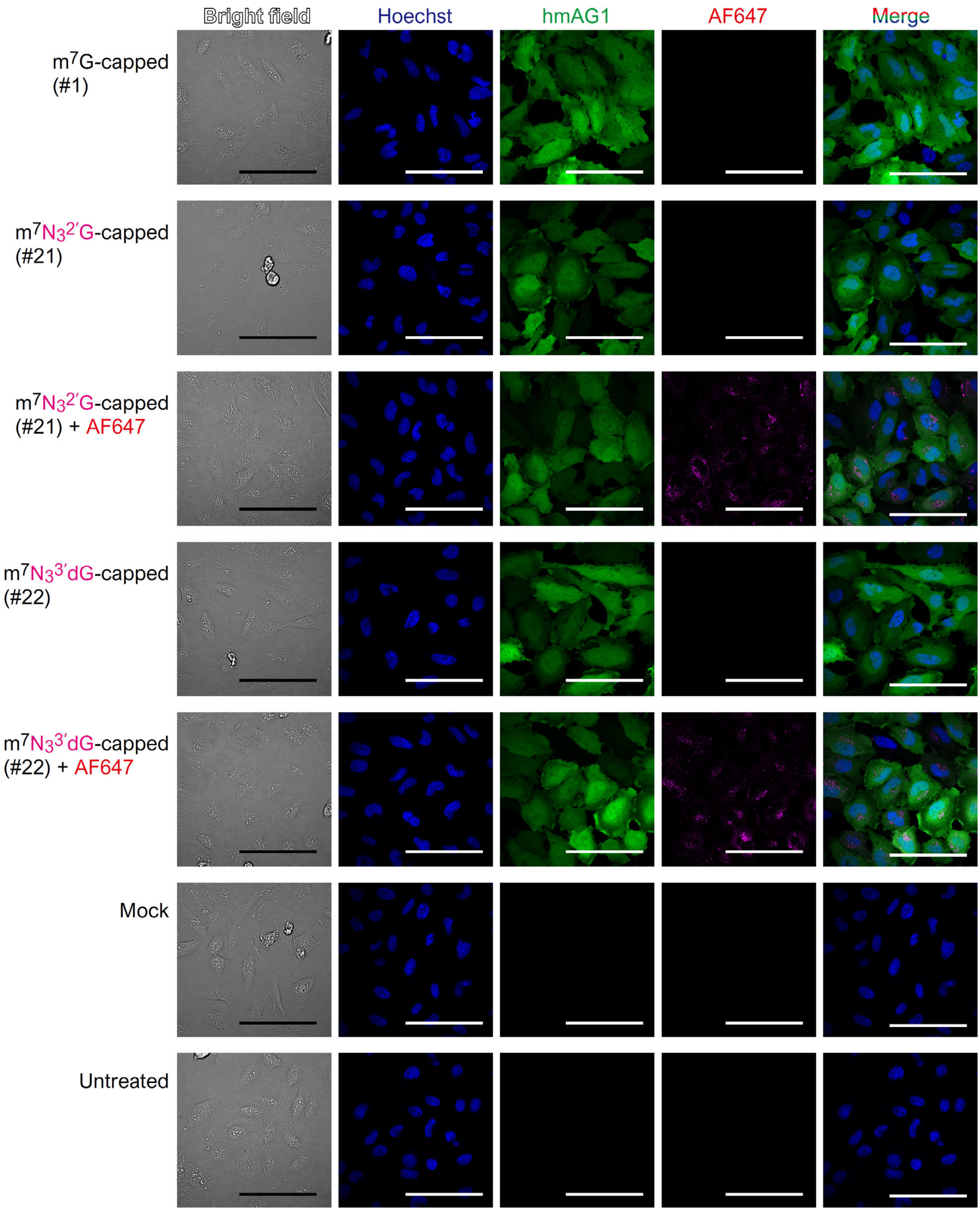

Supplementary Figure S11. Confocal microscopy of fluorescently labeled mRNA in HeLa cells. The left column shows bright field images, and the next three columns show fluorescent images for the nucleus (Hoechst), reporter protein (hmAG1) and RNA (AF647), respectively. The merged image of the three fluorescent images is shown in the next column. Scale bars, 100 µm. The rightmost column shows a magnification (100 µm×100 µm) of the boxed areas of the merged column. Mock: treated with transfection reagent without RNA; Untreated: not treated with a transfection reagent or RNA.

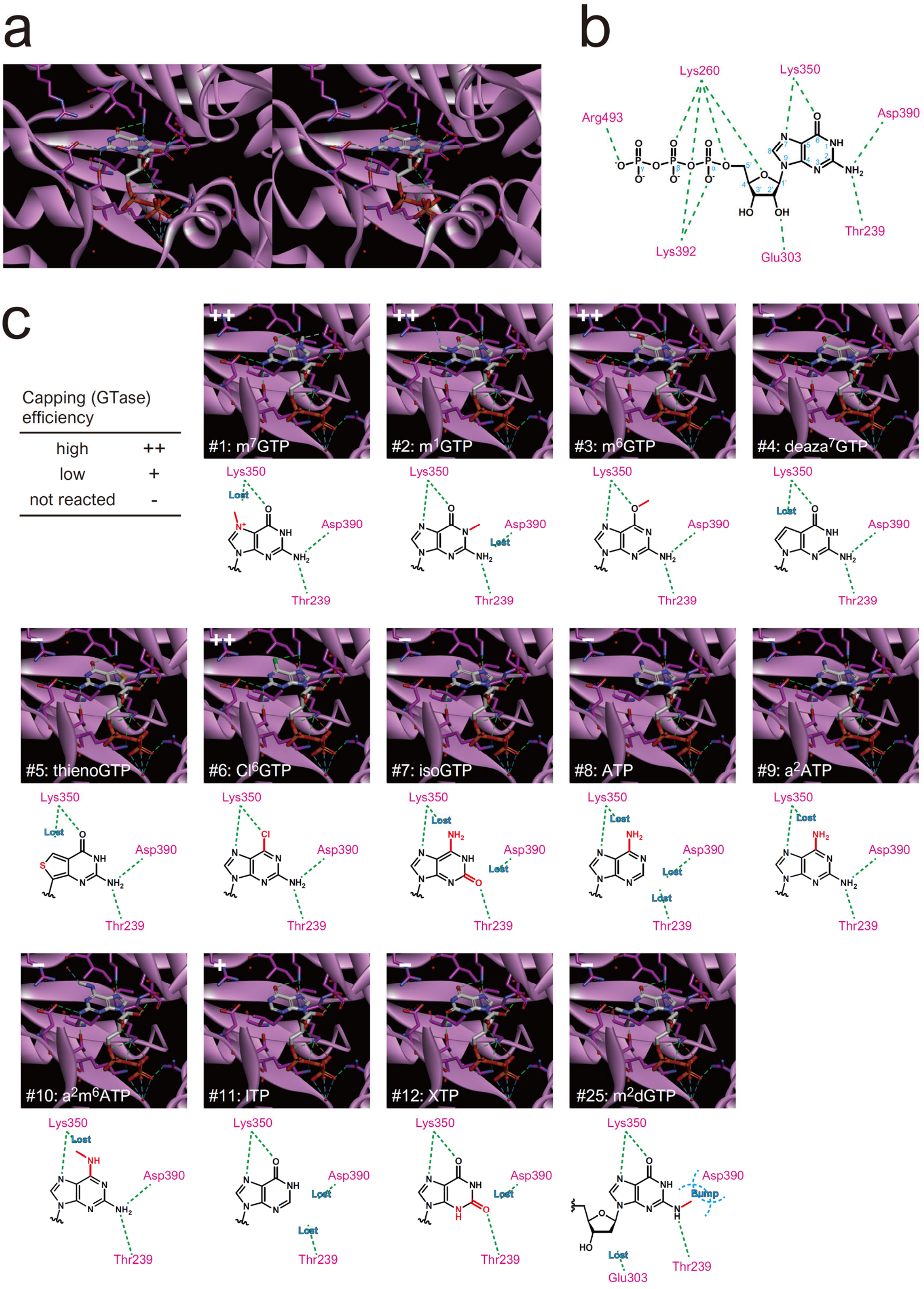

Supplementary Figure S12. Interaction between VCE and GTP. (a) Stereoscopic view of the guanylyltransferase active site in VCE (PDB ID: 4CKB). VCE is colored in purple, and GTP is colored in white (carbon atoms). Green dashed lines indicate hydrogen bonds. **(b)** Schema of hydrogen bonds (green) between GTP (black) and amino acid residues (purple) of VCE. **(c)** 3D models of VCE with each GTP analog.

Supplementary Table S1. Sequences of RNA used.

RNA name	Length (nt)	Sequence (5' -> 3')
5UTR(1-11)	11	GGGCGAAUUAA
hmAG1 (Azami-Green) mRNA	951	GGGCGAAUUAAGAGAGAAAAGAAGAGUAAGAAGAAAUAUAAGACACCGGUCGCCACCAUGGUGAGCGUGAUCAAGCCCGA<br>GAUGAAGAUCAAGCUGUGCAUGAGGGGCACCGUGAACGGCCACAACUUCGUGAUCGAGGGCGAGGGCAAGGGCAACCCCU<br>ACGAGGGCACCCAGAUCCUGGACCUGAACGUGACCGAGGGCGCCCCCCUGCCCUUCGCCUACGACAUCCUGACCACCGUG<br>UUCCAGUACGGCAACAGGGCCUUCACCAAGUACCCCGCCGACAUCCAGGACUACUUCAAGCAGACCUUCCCCGAGGGCUA<br>CCACUGGGAGAGGAGCAUGACCUACGAGGACCAGGGCAUCUGCACCGCCACCAGCAACAUCAGCAUGAGGGGCGACUGCU<br>UCUUCUACGACAUCAGGUUCGACGGCACCAACUUCCCCCCCAACGGCCCCGUGAUGCAGAAGAAGACCCUGAAGUGGGAG<br>CCCAGCACCGAGAAGAUGUACGUGGAGGACGGCGUGCUGAAGGGCGACGUGAACAUGAGGCUGCUGCUGGAGGGCGGCGG<br>CCACUACAGGUGCGACUUCAAGACCACCUACAAGGCCAAGAAGGAGGUGAGGCUGCCCGACGCCCACAAGAUCGACCACA<br>GGAUCGAGAUCCUGAAGCACGACAAGGACUACAACAAGGUGAAGCUGUACGAGAACGCCGUGGCCAGGUACUCCAUGCUG<br>CCCAGCCAGGCCAAGUGAAUCUAGACCUUCUGCGGGGCUUGCCUUCUGGCCAUGCCCUUCUUCUCUCCCUUGCACCUGUA<br>CCUCUUGGUCUUUGAAUAAAGCCUGAGUAGGAAAAAAAAAAAAAAAAAAAAAAAAAAAAAAAAAAAAAAAAAAAAAAAAA<br>AAAAAAAAAAAAAAAAAAAAAAAAAAAAAAAAAAAAAAAAAAAAAAAAAAAAAAAAAAAAAAAAAAAAAAA
iRFP670 mRNA	1,229	GGGCGAAUUAAGAGAGAAAAGAAGAGUAAGAAGAAAUAUAAGACACCGGUCGCCACCAUGGCGCGUAAGGUCGAUCUCAC<br>CUCCUGCGAUCGCGAGCCGAUCCACAUCCCCGGCAGCAUUCAGCCGUGCGGCUGCCUGCUAGCCUGCGACGCGCAGGCGG<br>UGCGGAUCACGCGCAUUACGGAAAAUGCCGGCGCGUUCUUUGGACGCGAAACUCCGCGGGUCGGUGAGCUACUCGCCGAU<br>UACUUCGGCGAGACCGAAGCCCAUGCGCUGCGCAACGCACUGGCGCAGUCCUCCGAUCCAAAGCGACCGGCGCUGAUCUU<br>CGGUUGGCGCGACGGCCUGACCGGCCGCACCUUCGACAUCUCACUGCAUCGCCAUGACGGUACAUCGAUCAUCGAGUUCG<br>AGCCUGCGGCGGCCGAACAGGCCGACAAUCCGCUGCGGCUGACGCGGCAGAUCAUCGCGCGCACCAAAGAACUGAAGUCG<br>CUCGAAGAGAUGGCCGCACGGGUGCCGCGCUAUCUGCAGGCGAUGCUCGGCUAUCACCGCGUGAUGUUGUACCGCUUCGC<br>GGACGACGGCUCCGGGAUGGUGAUCGGCGAGGCGAAGCGCAGCGACCUCGAGAGCUUUCUCGGUCAGCACUUUCCGGCGU<br>CGCUGGUCCCGCAGCAGGCGCGGCUACUGUACUUGAAGAACGCGAUCCGCGUGGUCUCGGAUUCGCGCGGCAUCAGCAGC<br>CGGAUCGUGCCCGAGCACGACGCCUCCGGCGCCGCGCUCGAUCUGUCGUUCGCGCACCUGCGCAGCAUCUCGCCCUGCCA<br>UCUCGAAUUUCUGCGGAACAUGGGCGUCAGCGCCUCGAUGUCGCUGUCGAUCAUCAUUGACGGCACGCUAUGGGGAUUGA<br>UCAUCUGUCAUCAUUACGAGCCGCGUGCCGUGCCGAUGGCGCAGCGCGUCGCGGCCGAAAUGUUCGCCGACUUCUUAUCG<br>CUGCACUUCACCGCCGCCCACCACCAACGCAGAUCUCAUAUGCAUCUCGAGUGAUAGUCUAGACCUUCUGCGGGGCUUGC<br>CUUCUGGCCAUGCCCUUCUUCUCUCCCUUGCACCUGUACCUCUUGGUCUUUGAAUAAAGCCUGAGUAGGAAAAAAAAAAA<br>AAAAAAAAAAAAAAAAAAAAAAAAAAAAAAAAAAAAAAAAAAAAAAAAAAAAAAAAAAAAAAAAAAAAAAAAAAAAAAAA<br>AAAAAAAAAAAAAAAAAAAAAAAAAAAAA

Cyan: 5′ UTR, green/red: protein-coding region (hmAG1 or iRFP670), orange: 3′ UTR, purple: poly(A) tail, start/stop codon: underlined.

Supplementary Table S2. Sequences of DNA used.

DNA name	Length (nt)	Sequence (5' -> 3')
5UTR(1-11) Sense strand	31	cagtgaattg*TAATACGACTCACTATA*GGGC
5UTR(1-11) Antisense strand	38	TTAATTCGCCC*TATAGTGAGTCGTATTA*caattcactg
Forward primer for mRNA	51	attg*TAATACGACTCACTATA*GGGCGAATTAAGAGAGAAAAGAAGAGTAAG
Reverse primer for mRNA	139	TTTTTTTTTTTTTTTTTTTTTTTTTTTTTTTTTTTTTTTTTTTTTTTTTTTTTTTTTTTTTTTTTTTTTTTTTTTTTTTT<br>TTTTTTTTTTTTTTTTTTTTTTTTTTTTTTTTTTTTTTTTCCTACTCAGGCTTTATTCA

Colored regions correspond to RNA in Supplementary Table S1. Italics: T7 promoter.

Supplementary Table S3. Primer sequences for real-time qPCR.

Primer	Length (nt)	Sequence (5' -> 3')	Tm (°C)	Amplicon size (bp)
Forward primer for hmAG1	20	GAGAGGAGCATGACCTACGA	53.6	145
Reverse primer for hmAG1	20	TCAGGGTCTTCTTCTGCATC	51.9	
Forward primer for GAPDH	23	GGTGGTCTCCTCTGACTTCAACA	55.6	79
Reverse primer for GAPDH	19	GTGGTCGTTGAGGGCAATG	52.2	
